# Regulation of Klotho Production by Mineralocorticoid Receptor Signaling in Renal Cell Lines

**DOI:** 10.3390/biom15111509

**Published:** 2025-10-25

**Authors:** Elena Kohm, Martina Feger, Michael Föller

**Affiliations:** Department of Physiology, University of Hohenheim, Garbenstraße 30, 70599 Stuttgart, Germany

**Keywords:** Klotho, vitamin D, aging, fibrosis, inflammation

## Abstract

Through the mineralocorticoid receptor, aldosterone controls extracellular volume and arterial blood pressure by stimulating Na^+^ absorption and K^+^ secretion in epithelial cells of the kidney, colon, and several glands. Hyperaldosteronism promotes fibrosis and inflammation in epithelial and non-epithelial tissues, thereby favoring loss of kidney and heart function. Mineralocorticoid receptor blockade therefore gains relevance especially in renal and cardiac disease. Kidney-derived Klotho is a powerful anti-aging protein with anti-fibrosis and anti-inflammatory effects providing cardio- and nephroprotection. We wondered whether Klotho expression and production is influenced by mineralocorticoid receptor agonists and antagonists. Using four renal cell lines, Madin-Darby canine kidney (MDCK), normal rat kidney, subtype 52E (NRK-52E), human kidney 2 (HK2) cells, and primary renal proximal tubule epithelial cells (RPTECs), and the four most frequently prescribed mineralocorticoid receptor blockers, spironolactone, eplerenone, finerenone, and esaxerenone, we assessed *Klotho* gene expression by qRT-PCR and Klotho protein by Western blotting. Aldosterone and eplerenone did not significantly affect *Klotho* expression in either cell line. Spironolactone enhanced *Klotho* expression in MDCK and NRK-52E cells and downregulated Klotho in HK2 cells and RPTECs. Novel non-steroidal mineralocorticoid receptor antagonist finerenone downregulated *Klotho* expression in MDCK, NRK-52E, and low-dose finerenone in HK2 cells. To conclude, common mineralocorticoid receptor antagonists are characterized by highly diverse effects on Klotho in four renal cell lines. Further studies are needed to define the role of mineralocorticoid receptor blockade for Klotho production.

## 1. Introduction

Aldosterone is the most significant mineralocorticoid in humans and produced in the adrenal cortex [1,2,3]. Classically and as suggested by their name, mineralocorticoids are implicated in the regulation of Na^+^ and K^+^ homeostasis [4,5], thereby impacting extracellular volume and arterial blood pressure [6,7]. These effects are mediated by binding of aldosterone to the mineralocorticoid receptor in different epithelial tissues, including renal collecting duct [8,9], salivary and sweat glands, or colon [10,11,12]. Consequently, transcription of the epithelial Na^+^ channel (ENaC) and different K^+^ channels is enhanced, resulting in absorption of Na^+^ and osmotically obliged water as well as K^+^ secretion [13,14].

Mineralocorticoid receptor blockade is a relatively old therapeutical approach introduced in the sixties when spironolactone became available to treat edema due to heart or renal failure [15]. Particularly as a consequence of the development of loop diuretics with markedly stronger diuretic effects than spironolactone, mineralocorticoid antagonist therapy seemed to be obsolete [16]. In addition, spironolactone also blocks the androgen receptor under therapeutic dosage, causing adverse effects such as gynecomastia [17]. Conversely, the affinity of spironolactone for the androgen receptor can therapeutically be exploited in hirsutism, i.e., excessive body hair in females, as this is dependent on androgen receptors [18]. Later, eplerenone with less antiandrogen effects was introduced [15].

It was due to a landmark trial published in 1999 that low-dose spironolactone treatment in addition to loop diuretics has become standard of care in the treatment of severe heart failure ever since, due to highly beneficial effects on morbidity and death [19]. Basic research later uncovered potent anti-fibrosis [20], anti-inflammatory [21], or anti-oxidative effects [22] of mineralocorticoid receptor blockade in non-epithelial tissues which may not only be beneficial in heart failure, but also in chronic kidney disease (CKD) [23]. However, the risk of severe hyperkalemia limited the use of spironolactone (and eplerenone) especially in CKD [24,25].

Finerenone is a novel mineralocorticoid antagonist with non-steroidal structure and anti-fibrosis and anti-inflammatory effects at least comparable to spironolactone [26,27], but with a considerably lower risk of hyperkalemia [28]. It is approved for and has beneficial effects in both CKD [29,30] and heart failure with preserved or mildly reduced ejection fraction [31]. The better safety profile with concomitant potent anti-fibrosis and anti-inflammatory effects is attributed to the recruitment of cofactors upon binding to the mineralocorticoid receptor different from those recruited by spironolactone or eplerenone [32,33].

Klotho is a renal protein expressed in the membrane serving as a co-receptor for fibroblast growth factor 23 (FGF23) [34,35,36]. Jointly, they regulate phosphate excretion and calcitriol (1,25(OH)_2_D_3_) synthesis [37,38]. Soluble Klotho is generated upon cleavage of transmembrane Klotho and has powerful anti-fibrosis [39], anti-inflammatory [40], or anti-oxidative effects [41] in various organs. Particularly owing to these features, Klotho is cardio-, neuro-, and renoprotective [42]. Moreover, Klotho overexpression results in profound life elongation, whereas lack of Klotho leads to rapid aging, suggesting that Klotho is a powerful anti-aging protein [42,43,44].

Research into the regulation of Klotho by aldosterone has yielded different results in cell culture and in vivo studies [45,46,47,48]. Due to these conflicting results and since a head-to-head comparison of the effects of the four most common mineralocorticoid receptor antagonists on Klotho production has not been performed, yet, we conducted a cell culture study testing the effects of the four most common mineralocorticoid blockers, spironolactone, eplerenone, finerenone, and esaxerenone, on Klotho in different renal cell lines.

## 2. Materials and Methods

### 2.1. Cell Culture

Madin-Darby canine kidney (MDCK) cells (CCL-34, ATCC, Manassas, VA, USA) were cultured at 37 °C and 5% CO_2_ in Dulbecco’s Modified Eagle Medium: Nutrient Mixture F-12 (DMEM/F-12; Gibco, Life Technologies, Thermo Fisher Scientific, Darmstadt, Germany) supplemented with 5% fetal bovine serum (FBS; Gibco), 2 mM L-glutamine (Gibco), 100 U/mL penicillin, and 100 μg/mL streptomycin (Gibco). Normal rat kidney, subtype 52E (NRK-52E) cells (CRL-1571, ATCC) were cultured at 37 °C and 5% CO_2_ in DMEM (Gibco) with 5% newborn calf serum (NBCS; Gibco), 100 U/mL penicillin, and 100 μg/mL streptomycin. Human kidney 2 (HK2) cells (CRL-2190, ATCC) were cultured at 5% CO_2_ and 37 °C in DMEM with 10% FBS, 100 U/mL penicillin, and 100 μg/mL streptomycin. hTERT-immortalized primary renal proximal tubule epithelial cells (RPTEC/TERT1; CRL-4031, ATCC) were cultured under standard cell culture conditions in DMEM/F-12 with 2.5 mM L-glutamine and 15 mM HEPES (Gibco) supplemented with the hTERT immortalized RPTEC growth kit (ATCC-ACS-4007, ATCC), G418 solution (G418-RO, Sigma-Aldrich, Schnelldorf, Germany), 100 U/mL penicillin, and 100 μg/mL streptomycin. Cells were seeded into 6-well or 12-well plates (Greiner Bio-One, Frickenhausen, Germany) and incubated for 24 h (MDCK, NRK-52E, HK2) or up to 85% confluency (RPTECs), followed by a 24-h exposure to different concentrations of aldosterone (A9477, Sigma-Aldrich), or 12 h up to 48 h of exposure to spironolactone (J60119.03, Thermo Fisher Scientific), eplerenone (S1707, Selleck Chemicals, Houston, TX, USA), finerenone (S9702, Selleck Chemicals) or esaxerenone (TGM-T15246; Biomol, Hamburg, Germany). In addition, 24-h treatments with testosterone (T1500, Sigma-Aldrich), progesterone (P8783, Sigma-Aldrich), flutamide (4094, Tocris, Bio-Techne, Wiesbaden, Germany), or mifepristone (1479, Tocris) were carried out. Furthermore, aldosterone exposure for 24 h was carried out in cells cultured in growth medium with 5% charcoal-stripped FBS (Gibco) instead of standard FBS. For all treatments, control cells were incubated with the appropriate volume of vehicle (dimethyl sulfoxide (DMSO; AppliChem, Darmstadt, Germany) or ethanol (Carl Roth, Karlsruhe, Germany)). A light microscope photograph of the cells is given in Supplementary Materials Figure S1.

### 2.2. Quantitative Real-Time Polymerase Chain Reaction

The isolation of total RNA from MDCK cells was accomplished by means of the RNA isolation kit NucleoSpin RNA from Machery-Nagel (Düren, Germany). Total RNA was isolated from NRK-52E and HK2 cells and from RPTECs based on phenol–chloroform extraction (TriFast Reagent, VWR, Bruchsal, Germany), and RNA (1.2 µg) was transcribed with the GoScript Reverse Transcription System using random primers (Promega, Mannheim, Germany) on a Biometra TAdvanced thermocycler (Analytik Jena, Jena, Germany). Quantitative real-time polymerase chain reaction (qRT-PCR) using 2 µL of cDNA was carried out on a CFX Connect Real-Time PCR Detection System (Bio-Rad Laboratories, Feldkirchen, Germany). Reaction mixes containing 0.25 μM (*Klotho*) or 0.5 μM (*TATA box-binding protein*; *TBP*) of each primer, 10 μL GoTaq qPCR Master Mix (Promega), and 6 or 7 µL water were used. QRT-PCR conditions were 95 °C for 2 min; 40 cycles of 95 °C for 10 s, primer-specific temperature for 30 s; and 72 °C for 25 s.

The following primers (5′ → 3′) and temperatures were used for qRT-PCR:

*Klotho* (*Kl*), 54 °C (rat): CAACTACATTCAAGTGGACC and CAGTAAGGTTTTCTCTTCTTGG;

*Klotho* (*KL*), 56 °C (dog): AAATGAAGCTCTGAAAGCC and AATGATAGAGGCCAAACTTC;

*Klotho* (*KL*), 59 °C (human): TGGAAACCTTAAAAGCCATCAAGC and CCACGCCTGATGCTGTAACC;

*Tbp*, 57 °C (rat): ACTCCTGCCACACCAGCC and GGTCAAGTTTACAGCCAAGATTCA;

*TBP*, 60 °C (dog): CCTATTACCCCTGCCACACC and GCTCCCGTACACACCATCTT;

*TBP*, 59 °C (human): TGCACAGGAGCCAAGAGTGAA and CACATCACAGCTCCCCACCA.

The relative mRNA transcription levels were calculated using the 2^−ΔCT^ method and *TBP* as the internal reference. Further information on the primersis given in Supplementary Materials Table S1 and Figure S2.

### 2.3. Qualitative Expression Analysis

Two µL of cDNA transcribed from DNase-treated RNA from MDCK and HK2 cells were used. Total RNA from a healthy dog uterus was extracted with the phenol–chloroform method and processed with the RNeasy Mini Kit and RNase-free DNase Set (both from Qiagen, Hilden, Germany) according to the manufacturer’s protocol and transcribed with the GoScript Reverse Transcription System using random primers (Promega). Two µL of cDNA were used. The cDNA from NRK-52E cells was used without prior DNase treatment. PCR reaction mixes contained 0.25 or 0.5 µM of each primer, 10 μL GoTaq Green Master Mix (Promega), and water up to a total volume of 20 µL. PCR conditions were 94 °C for 3 min; 30 cycles of 94 °C for 30 s, primer-specific temperature for 30 s, 72 °C for 20 s; and 72 °C for 5 s.

The following primers (5′ → 3′) and temperatures were utilized:

*Nr3c2*, 59 °C (rat): TCCAGAAAACGTGTCAAGCTCT and CCCTTCCACGGCTCTTTTGA;

*NR3C2*, 56 °C (dog): TTGCCTTGAGCTGGAGATCG and GCCGTCCTTTGGAATTGTGC;

*NR3C2*, 58 °C (human): CCAGGATTTAAAAACTTGCC and CATTAAAGACTAGGTCTGGTG;

*Ar*, 59 °C (rat): TCCGGACCTTATGGGGACAT and ACTTCTGTTTCCCTTCCGCA;

*AR*, 59 °C (dog): GGGGATCTGTAGCCCCCTAT and CTGGCAGTCTCCAAACGCAT;

*AR*, 60 °C (human): CCTGATGTGTGGTACCCTGG and GCAGTCTCCAAACGCATGTC;

*Pgr*, 59 °C (rat): ATGGTCCTTGGAGGTCGTAAG and AGGTTGATGAGTGGCGGAAC;

*PGR*_1, 59 °C (dog): GTACCAGCCGTACCTCAACTA and AATAGTTATGCTGCCCTTCCA;

*PGR*_2, 55 °C (dog): GTGTACCAGCCGTACCTCA and ATAGTTATGCTGCCCTTCCATT;

*PGR*_3, 59 °C (dog): GGTGTACCAGCCGTACCTCAAC and ACTTTTTAAATTTTCGACCTCCAAGG;

*PGR*_4, 59 °C (dog): ATGGTGTCCTAACTTGTGG and ACAATGCAGTCATTTCTTCC;

*PGR,* 61 °C (human): AGGTCTACCCGCCCTATCTC and AGTAGTTGTGCTGCCCTTCC.

PCR amplificates were loaded onto 1.5% agarose gels. Additionally, qRT-PCR was performed. Reaction mixes contained 0.25 μM or 0.5 μM of each primer, 10 μL GoTaq qPCR Master Mix (Promega), and 6 or 7 μL water. QRT-PCR conditions were 95 °C for 2 min; 40 cycles of 95 °C for 10 s, primer-specific temperature for 30 s; and 72 °C for 25 s.

### 2.4. Western Blotting

HK2 cells were seeded into T25 cell culture flasks (Greiner Bio-One) and RPTECs into 6-well plates and cultured for 24 h, followed by a 24-h treatment with 30 µM spironolactone or vehicle only. Cells were lyzed using ice-cold RIPA buffer (Cell Signaling Technology, Danvers, MA, USA) supplemented with additional protease and phosphatase inhibitor cocktail and EDTA (Halt, Thermo Fisher Scientific). Total protein concentration was measured by Bradford assay (Thermo Fisher Scientific). Thirty µg (or 40 µg) protein were subjected to 10% SDS-PAGE and standard Western blotting. The following antibodies were used: rat anti-human Klotho monoclonal antibody (Clone No. KM2076; Hölzel Diagnostika, Köln, Germany), rabbit anti-GAPDH (D16H11) XP monoclonal antibody (Cell Signaling Technology), goat anti-rat (Novus Biologicals, Bio-Techne, Wiesbaden, Germany) and goat anti-rabbit (Cell Signaling Technology) IgG, HRP-linked antibody. Proteins were visualized using Clarity Western (Bio-Rad Laboratories) or Westar Hypernova (Cyanagen, Bologna, Italy) ECL substrate. Protein bands were detected by ChemiDoc MP Imaging System (Bio-Rad Laboratories) and signal intensities were determined using Image Lab Software (version 6.1, Bio-Rad Laboratories). The data are shown as ratio of Klotho over loading control glyceraldehyde-3-phosphate dehydrogenase (GAPDH).

### 2.5. Statistics

Data are given as arithmetic means ± standard error of the mean (SEM). *N* represents the number of independent experiments. Shapiro–Wilk test was used to test for normal distribution. Two groups were subjected to two-tailed paired *t*-test, more than two groups to repeated measures one-way analysis of variance (ANOVA) followed by Dunnett’s multiple comparisons test, Šidák’s multiple comparisons test or Friedman test. Differences were considered significant if *p* < 0.05. Statistics were analyzed using GraphPad Prism version 10.3.1 (GraphPad Software, Boston, MA, USA).

## 3. Results

**Figure 1:**
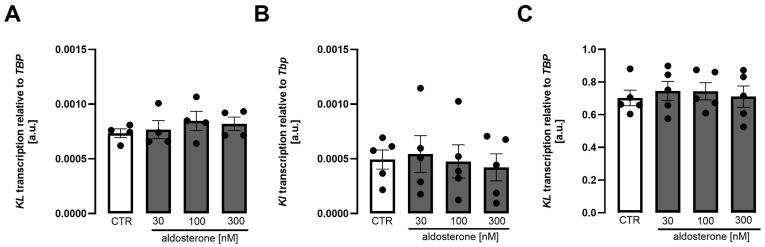
*Klotho* expression is not significantly regulated by aldosterone in MDCK, NRK-52E, and HK2 cells. Arithmetic means ± SEM of rel. *Klotho* gene expression in MDCK ((**A**), n = 4), NRK-52E ((**B**), n = 5), and HK2 ((**C**), n = 5) cells treated with or without aldosterone for 24 h. One-way ANOVA followed by Dunnett’s multiple comparisons or Friedman test. A.u., arbitrary units.

Our study aimed to identify the relevance of mineralocorticoid receptor agonism and antagonism for Klotho production. As a first step, we studied the expression of mineralocorticoid receptor (encoded by *NR3C2*), androgen receptor (encoded by *AR*), and progesterone receptor (encoded by *PGR*) in three renal cell lines, Madin-Darby canine kidney (MDCK) cells, normal rat kidney, subtype 52E (NRK-52E) cells, and human kidney 2 (HK2) cells. As illustrated in Supplementary Materials Figure S3, mineralocorticoid receptor and androgen receptor expression could be verified in all three cell lines, but progesterone receptor could only be detected in NRK-52E and HK2 cells, not in MDCK cells, despite employing four different primer pairs. Next, we exposed the cells to increasing concentrations of aldosterone for 24 h and estimated *Klotho* gene expression by qRT-PCR. According to Figure 1, aldosterone did not significantly affect *Klotho* mRNA abundance in MDCK cells (Figure 1A), NRK-52E cells (Figure 1B), and HK2 cells (Figure 1C). The lack of an aldosterone effect on *Klotho* may have been due to already saturating aldosterone concentrations in normal FBS used for cell culture. In order to test this possibility, the experiment was repeated with charcoal-stripped FBS. Again, aldosterone did not significantly affect *Klotho* expression (Supplementary Materials Figure S4).

**Figure 2:**
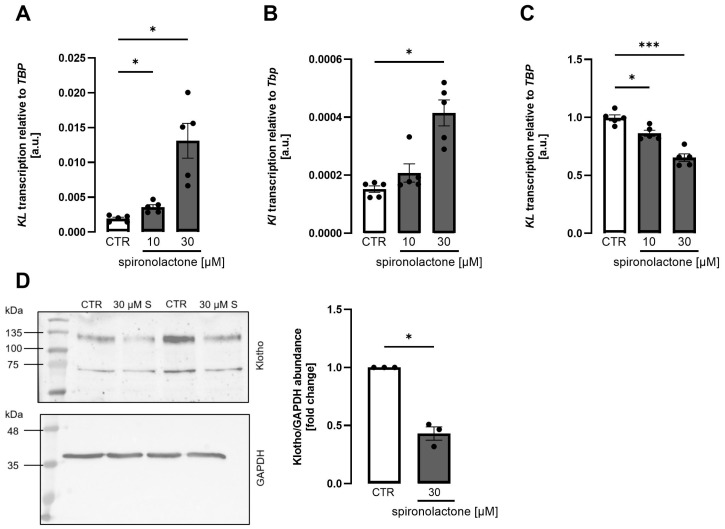
*Klotho* expression is upregulated by spironolactone in MDCK and NRK-52E cells but downregulated in HK2 cells. Arithmetic means ± SEM of rel. *Klotho* gene expression in MDCK ((**A**), n = 5), NRK-52E ((**B**), n = 5), and HK2 ((**C**), n = 5) cells treated with or without spironolactone for 24 h. Representative Western blot (left panel) and densitometric analysis of Klotho protein expression (right panel, n = 3) in HK2 cells treated with or without spironolactone (S) for 24 h (**D**). Note the Klotho band in the range of 120 kDa. The original Western blot images are shown in Supplementary Materials Figure S6. One-way ANOVA followed by Dunnett’s multiple comparisons or Friedman test and two-tailed paired *t*-test; * *p* < 0.05, *** *p* < 0.001. A.u., arbitrary units.

Spironolactone is still the most frequently prescribed mineralocorticoid antagonist, in particular in heart failure. We next explored whether spironolactone affects Klotho production. In both MDCK (Figure 2A) and NRK-52E (Figure 2B) cells, spironolactone dose-dependently up-regulated *Klotho* gene expression, as assessed by qRT-PCR. In HK2 cells, however, spironolactone dose-dependently decreased both, *Klotho* mRNA expression (Figure 2C) and Klotho protein abundance (Figure 2D) estimated from Western blotting. The time dependence of the effect is displayed in Supplementary Materials Figure S5.

**Figure 3:**
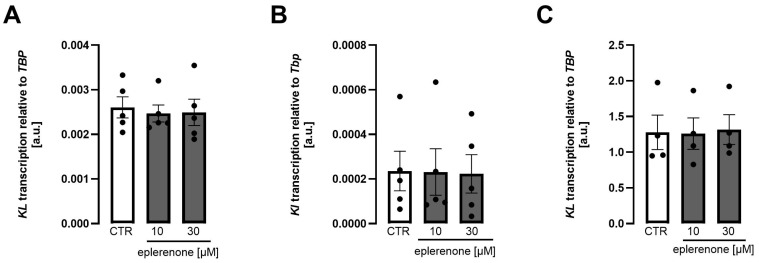
*Klotho* expression is not significantly regulated by eplerenone in MDCK, NRK-52E, and HK2 cells. Arithmetic means ± SEM of rel. *Klotho* gene expression in MDCK ((**A**), n = 5), NRK-52E ((**B**), n = 5), and HK2 ((**C**), n = 4) cells treated with or without eplerenone for 24 h. One-way ANOVA followed by Dunnett’s multiple comparisons or Šidák’s multiple comparisons test. A.u., arbitrary units.

Eplerenone is newer than spironolactone and more specific for the mineralocorticoid receptor than spironolactone, which exhibits appreciable affinity for other receptors, particularly the androgen receptor [15]. In contrast to spironolactone, similar concentrations of eplerenone did not significantly affect *Klotho* gene expression in either renal cell line, MDCK cells (Figure 3A), NRK-52E cells (Figure 3B), or HK2 cells (Figure 3C). The time dependence of the effect is displayed in Supplementary Materials Figure S7.

**Figure 4:**
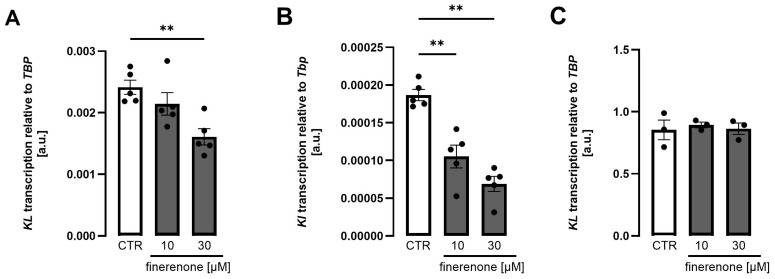
*Klotho* expression is downregulated by finerenone in MDCK and NRK-52E cells but not in HK2 cells. Arithmetic means ± SEM of rel. *Klotho* gene expression in MDCK ((**A**), n = 5), NRK-52E ((**B**), n = 5), and HK2 ((**C**), n = 3) cells treated with or without finerenone for 24 h. One-way ANOVA followed by Dunnett’s multiple comparisons test; ** *p* < 0.01. A.u., arbitrary units.

Finerenone belongs to a novel class of drugs termed non-steroidal mineralocorticoid blockers, characterized by high affinity for the mineralocorticoid receptor and low affinity for other receptors, but not being based on a steroid structure. According to Figure 4, finerenone, applied at comparable concentrations, dose-dependently reduced *Klotho* expression in both MDCK (Figure 4A) and NRK-52E (Figure 4B) cells without significantly affecting *Klotho* expression in HK2 cells (Figure 4C). The time dependence of the effect is displayed in Supplementary Materials Figure S8. At 1 µM, finerenone very moderately, but statistically significantly, reduced *Klotho* in all three cell lines (Supplementary Materials Figure S9A–C). Even at 30 µM, finerenone did not diminish viability in either cell line (Supplementary Materials Figure S9D). Novel aldosterone antagonist esaxerenone enhanced *Klotho* expression in MDCK cells whilst suppressing *Klotho* in NRK-52E cells and having no significant effect in HK2 cells (Supplementary Materials Figure S10).

Our experiments thus far revealed highly diverse effects of mineralocorticoid receptor manipulation in the three renal cell lines. Species differences may, at least in part, account for these differences. We therefore performed further experiments in a second human cell line of renal origin, RPTECs. As illustrated in Supplementary Materials Figure S11A, RPTECs expressed the mineralocorticoid receptor. Importantly, spironolactone suppressed *Klotho* gene expression (Supplementary Materials Figure S11B), whereas eplerenone (Supplementary Materials Figure S11C) and finerenone (Supplementary Materials Figure S11D) did not significantly alter it. Klotho protein expression was near the detection limit for Western blotting in RPTECs, whereas it could again be readily detected in HK2 cells which were included as a positive control (Supplementary Materials Figure S11). The Klotho band in the range of 120 kDa—visible in HK2 cells but absent in RPTECs—is indicated by a red arrow (Supplementary Materials Figure S11E).

**Figure 5:**
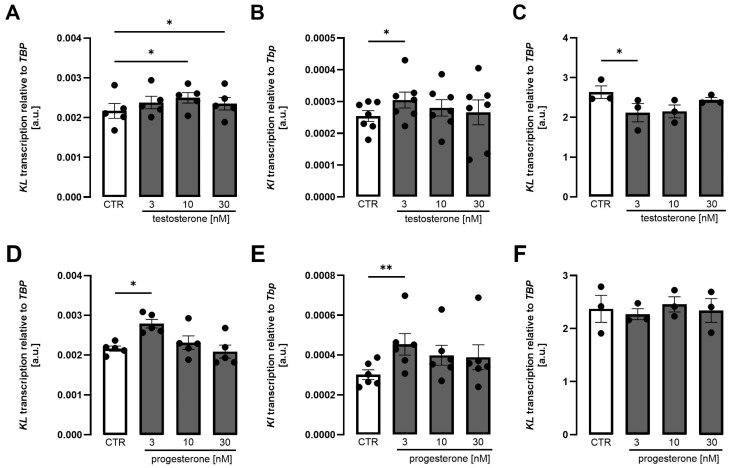
Testosterone and progesterone moderately increased *Klotho* expression in MDCK and NRK-52E cells. Arithmetic means ± SEM of rel. *Klotho* gene expression in MDCK ((**A**,**D**), n = 5), NRK-52E ((**B**), n = 7; (**E**), n = 6), and HK2 ((**C**,**F**), n = 3) cells treated with or without testosterone (**A**–**C**) or progesterone (**D**–**F**) for 24 h. One-way ANOVA followed by Dunnett’s multiple comparisons or Friedman test; * *p* < 0.05, ** *p* < 0.01. A.u., arbitrary units.

In addition to blocking mineralocorticoid receptor, spironolactone may evoke clinically relevant antiandrogen and anti-gestagen effects, causing common adverse effects typical of spironolactone therapy [49]. We therefore performed further experiments to investigate whether testosterone and progesterone impact *Klotho* expression. As illustrated in Figure 5, both testosterone (Figure 5A–C) and progesterone (Figure 5D–F) moderately enhanced *Klotho* expression in MDCK cells (Figure 5A,D) and NRK-52E cells (Figure 5B,E). Virtually no effect was seen in HK2 cells (Figure 5C,F).

**Figure 6:**
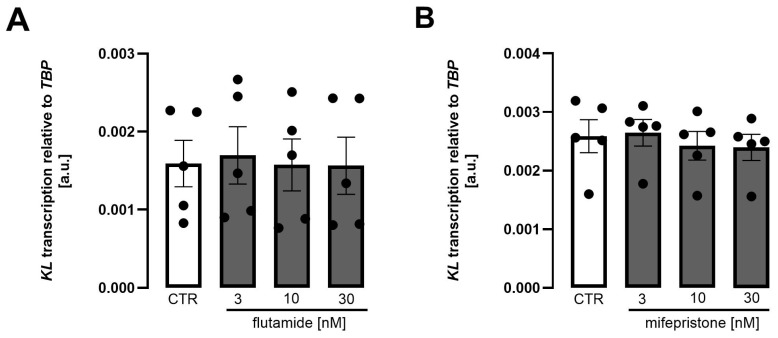
Testosterone antagonist flutamide and progesterone antagonist mifepristone did not significantly modify *Klotho* mRNA transcripts in MDCK cells. Arithmetic means ± SEM of rel. *Klotho* gene expression in MDCK cells treated with or without flutamide ((**A**), n = 5) or mifepristone ((**B**), n = 5) for 24 h. One-way ANOVA followed by Dunnett’s multiple comparisons test. A.u., arbitrary units.

The subtle, but statistically significant effects of testosterone and progesterone prompted us to perform further experiments with flutamide, an antiandrogen used in prostate cancer therapy [50], and mifepristone, an anti-gestagen used to end pregnancy [51]. It is shown in Figure 6 that neither flutamide (Figure 6A) nor mifepristone (Figure 6B) significantly affected *Klotho* expression in MDCK cells.

## 4. Discussion

According to our study, the role of mineralocorticoid receptor agonism and antagonism is highly diverse in the four renal cell lines investigated.

The first lesson is that aldosterone added to cell culture medium did not significantly modify *Klotho* gene expression in either cell line, whereas the antagonists exhibited different effects. Even under usage of charcoal-stripped FBS, aldosterone did not significantly affect *Klotho*. Therefore, aldosterone possibly present in ordinary FBS cannot explain this phenomenon.

Spironolactone dose-dependently induced *Klotho* expression in MDCK and NRK-52E cells. This finding is in line with an earlier report demonstrating upregulation of Klotho by spironolactone in HEK293 cells [45]. In view of the versatile health-beneficial effects of Klotho, spironolactone-dependent *Klotho* upregulation also makes perfectly sense as spironolactone has been shown to favorably influence heart disease and renal function at least in some models of kidney disease [25,52]. In contrast, Klotho was downregulated in HK2 cells following exposure to spironolactone on both mRNA and protein level. In RPTECs, however, Klotho protein could not be detected by Western blotting. The different origin of the four cell lines studied may help explain why the same antagonists exerted different effects on Klotho in the cell lines studied. Whereas HK2 cells [53], NRK-52E cells [54], and RPTECs [55] are derived from the renal proximal tubule, MDCK cells are distal tubular cells [56]. Also differences in cellular mineralocorticoid receptor levels or coactivators and corepressors may be relevant [57,58]. Also, species differences may account for the observed diverse effects on Klotho. In line with this, the three antagonists spironolactone, eplerenone, and finerenone caused the same effects in the two human cell lines studied.

Interestingly, the two other mineralocorticoid blockers tested, eplerenone and finerenone, markedly differed from spironolactone with regard to their respective effects on *Klotho* expression: eplerenone did not significantly affect *Klotho* in any cell line. Finerenone, however, suppressed *Klotho* expression in MDCK, NRK-52E, and HK2 cells at lower concentrations.

Differences between steroidal mineralocorticoid antagonists spironolactone and eplerenone, on the one hand, and the novel non-steroidal blocker finerenone are well documented [32]. Finerenone is most specific for the mineralocorticoid receptor, exhibiting the lowest IC50 value for the mineralocorticoid receptor and the highest for other steroid hormone receptors including androgen, gestagen, and glucocorticoid receptor [27]. Moreover, finerenone may also differ from spironolactone and eplerenone in the signaling downstream of binding of the antagonist to the mineralocorticoid receptor [32,33,59]; particularly, it induces a pattern of cofactor binding to the mineralocorticoid receptor different from that of the other two blockers [60], resulting in more powerful cardiac anti-fibrosis effects [33]. These properties may also account, at least partially, for the different effects of finerenone compared to the other antagonists. Notably, finerenone did not compromise cell viability.

Further significant differences exist between spironolactone and eplerenone. Spironolactone exhibits high affinity for the mineralocorticoid receptor, but almost similar affinity for the androgen receptor and a little less for the gestagen receptor [15,61]. Conversely, eplerenone has no appreciable affinity for other steroid receptors, but also markedly lower affinity for the mineralocorticoid receptor [15,62]. These facts are in line with eplerenone not significantly influencing *Klotho* at concentrations at which both spironolactone and finerenone were elucidated to significantly affect *Klotho*.

Due to spironolactone’s unique and clinically relevant antagonism of androgen and progesterone receptor, we also performed experiments with androgen and gestagen receptor agonists and antagonists. These experiments revealed subtle, but statistically significant, positive effects of testosterone and progesterone on *Klotho* expression in MDCK and NRK-52E cells; however, testosterone antagonist flutamide and gestagen receptor antagonist mifepristone did not significantly modify *Klotho* expression. In HK2 cells, virtually no progesterone and testosterone effect on *Klotho* was observed. Therefore, it appears to be unlikely that androgen and progesterone receptor antagonism were of high relevance for the spironolactone effect on Klotho, although androgen receptor expression could be detected in all three cell lines and progesterone receptor expression in NRK-52E cells and HK2 cells. Although an earlier study described progesterone effects in MDCK cells, however, without expression data [63], we were not able to verify progesterone receptor expression using four different primer pairs and canine uterus as a positive control.

Undoubtedly, regulation of Klotho by commonly used mineralocorticoid receptor antagonists would be of high medical relevance. In view of the various and clinically significant beneficial effects of Klotho on the target organs of mineralocorticoid blockers, i.e., heart and kidney [42], upregulation of Klotho by mineralocorticoid blockers would be desirable and could, at least in theory, contribute to the anti-fibrosis and anti-inflammatory effects of these drugs on the heart and kidney [39,40]. The highly diverse effects of the different antagonists on Klotho may, at least in part, be explained by mineralocorticoid receptor-independent effects. As a matter of fact, the antagonists differ in their affinity for the mineralocorticoid receptor [64]. Although our study was not able to fully explain the reason for the opposing effects, these may still be of clinical interest. In particular, since it is well conceivable that these effects are, at least partly, independent of the mineralocorticoid receptor, the lack of an aldosterone effect on Klotho does not preclude a relevance of the antagonists for Klotho.

It is a clear limitation of our study that it lacks in vivo data which are necessary to interpret the relevance of mineralocorticoid receptors for Klotho. In addition, care has to be taken when interpreting data obtained in NRK-52E cells that *Klotho* expression is rather low, with cycle threshold (Ct) values being in the range of app. 32–34. Also, it would have been advisable for our gene expression analysis to have based it on at least two reference genes. Moreover, our study underscores the need to carefully interpret cell culture data in view of the partly opposing effects obtained in four different renal cell lines used for Klotho research. The partly opposing effects observed in our study may have also been affected by the different culture conditions required by the four cell lines studied.

## 5. Conclusions

Our study uncovers the different actions of three widely used mineralocorticoid receptor antagonists on *Klotho* expression in different renal cell lines. Additional in vivo experiments are needed to further substantiate the effect of mineralocorticoid receptor modulation on Klotho.

## Data Availability

The original contributions presented in this study are included in the article/Supplementary Materials. Further inquiries can be directed to the corresponding author.

## Supplementary Materials

The following supporting information can be downloaded at: https://www.mdpi.com/article/10.3390/biom15111509/s1, Figure S1: Light microscopy photographs of the renal cells; Figure S2: Evaluation of primer efficiency and RNA quality; Figure S3: Qualitative expression analysis of *NR3C2*, *AR*, and *PGR* in MDCK, NRK-52E, and HK2 cells; Figure S4: Aldosterone did not significantly affect *Klotho* expression in MDCK, NRK-52E, and HK2 cells cultured in growth medium supplemented with charcoal-stripped fetal bovine serum; Figure S5: Time dependence of the spironolactone effect on *Klotho* expression in MDCK, NRK-52E, and HK2 cells; Figure S6: Original Western blot shown in Figure 2D; Figure S7: Time dependence of the eplerenone effect on *Klotho* expression in MDCK, NRK-52E, and HK2 cells; Figure S8: Time dependence of the finerenone effect on *Klotho* expression in MDCK, NRK-52E, and HK2 cells; Figure S9: Low-dose finerenone reduced *Klotho* expression in MDCK, NRK-52E, and HK2 cells, and a high dose of finerenone had no effect on cell viability; Figure S10: Esaxerenone increased *Klotho* expression in MDCK cells, decreased *Klotho* in NRK-52E cells, and had no significant effect on *Klotho* in HK2 cells; Figure S11: Spironolactone reduced *Klotho* mRNA transcription in RPTECs; Figure S12: Original Western blot shown in Figure S11E; Table S1: Additional information on the primers used.

## Abbreviations

The following abbreviations are used in this manuscript:

CKD	Chronic kidney disease
DMEM/F-12	Dulbecco’s Modified Eagle Medium: Nutrient Mixture F-12
FBS	Fetal bovine serum
FGF23	Fibroblast growth factor 23
GAPDH	Glyceraldehyde-3-phosphate dehydrogenase
HK2	Human kidney 2
MDCK	Madin-Darby canine kidney
NBCS	Newborn calf serum
NRK-52E	Normal rat kidney, subtype 52E
RPTEC	Renal proximal tubule epithelial cell
TBP	TATA box-binding protein
