# Supplementary material for: Regulation of Klotho Production by Mineralocorticoid Receptor Signaling in Renal Cell Lines
Source: Biomolecules. 2025 Oct 25;15(11):1509. doi: 10.3390/biom15111509 (PMC12650181; doi:10.3390/biom15111509)
Supplement: Supplementary file 1 [file biomolecules-15-01509-s001.zip › biomolecules-3623839-supplementary-ori figure.pdf]

Images of original drafts

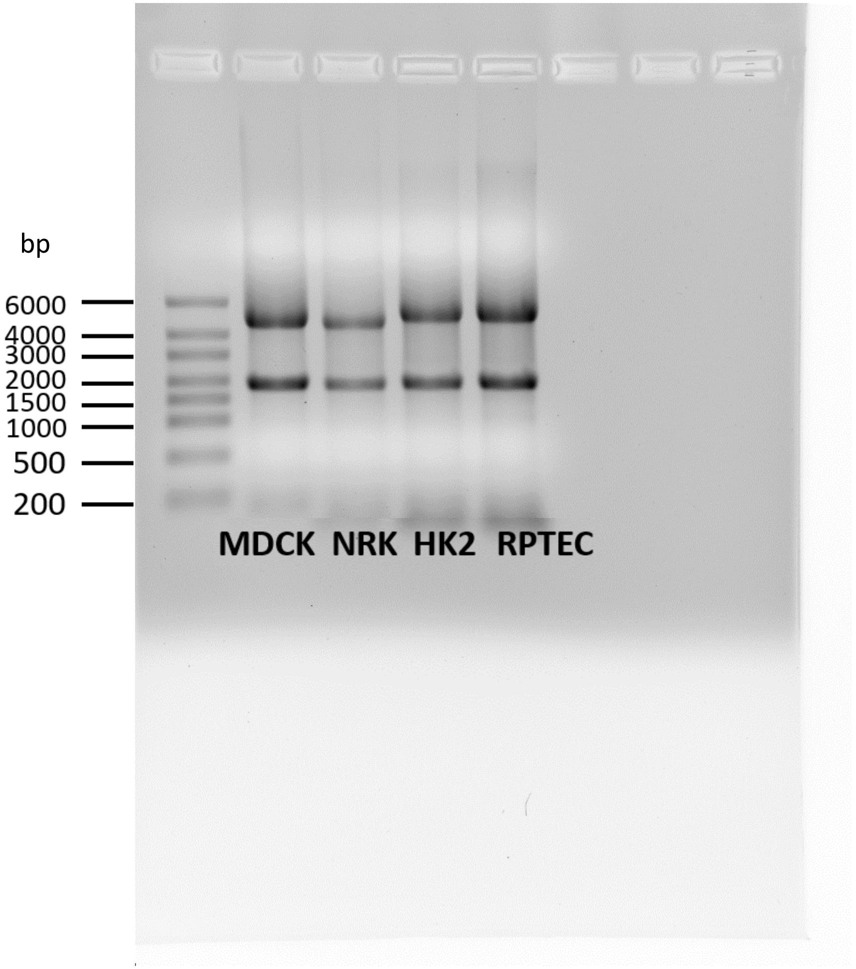

Original gel image shown in Suppl. Figure S2 D. Qualitative detection of 18S rRNA and 28S rRNA in MDCK, NRK-52E, HK2 and RPTEC cells on 1% Agarose gel contaning formaldehyde and ethidium bromide.

Images of original drafts

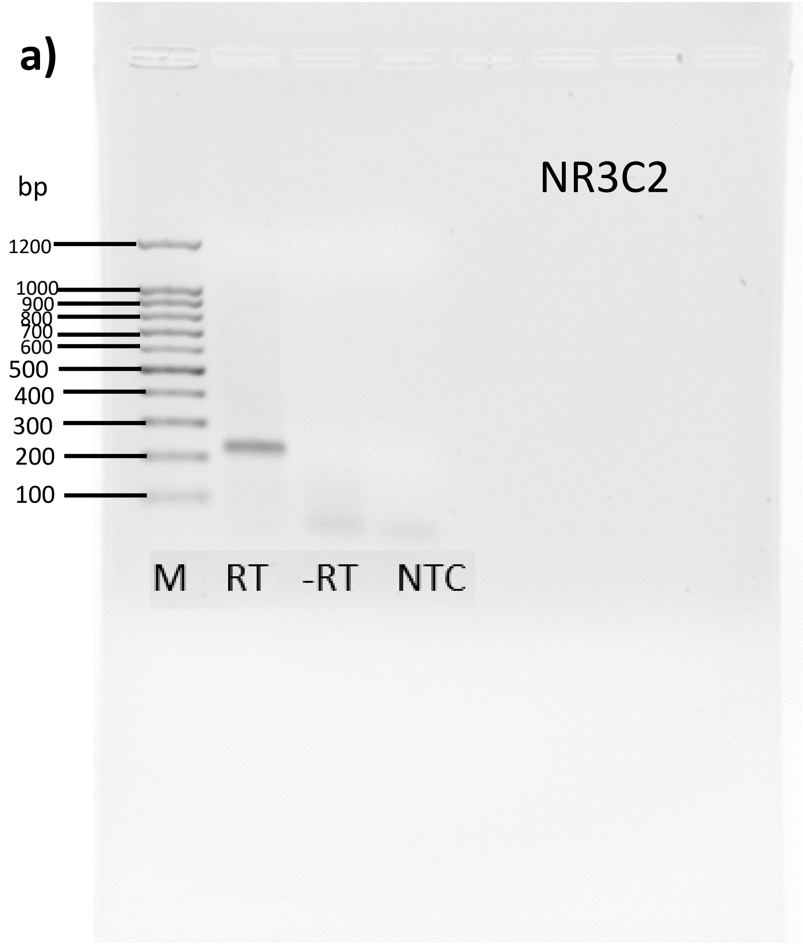

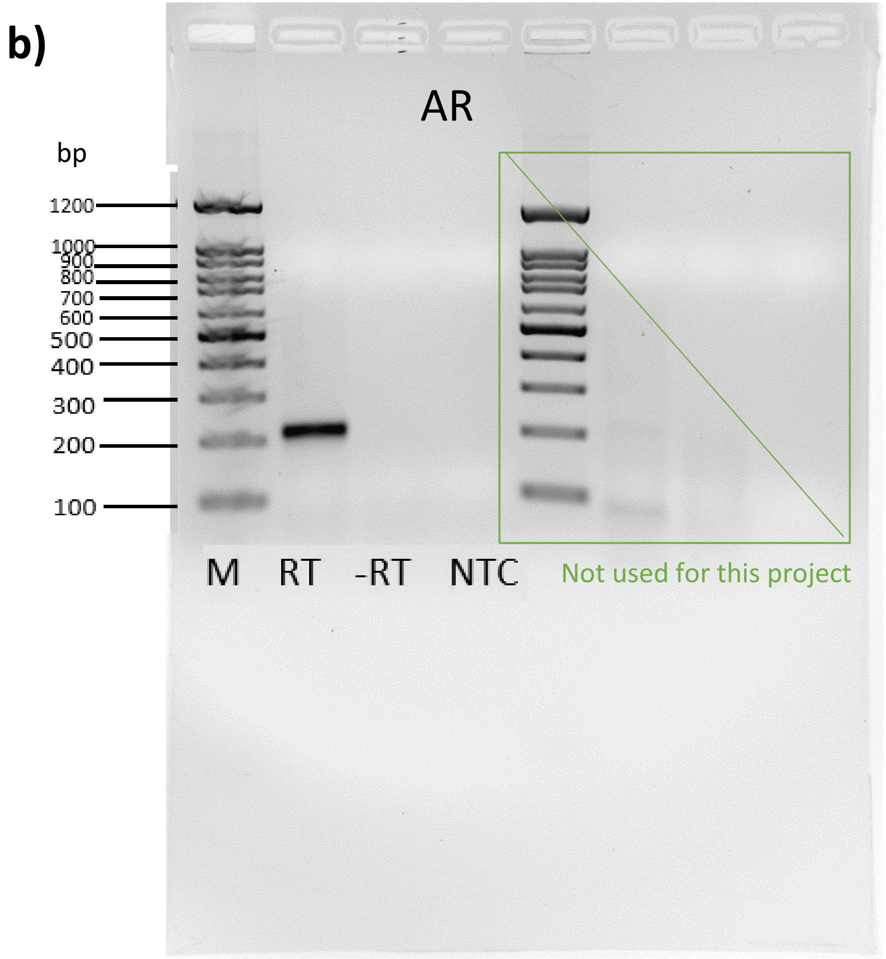

Original gel image shown in Suppl. Figure S3 A. a) Qualitative detection of NR3C2 in MDCK cells. Amplification products after qPCR on a 1.5% agarose gel. Amplicons of reverse transcriptase (RT), without reverse transcriptase (-RT) and a non target control (NTC) were applied. **b)** Qualitative detection of AR in MDCK cells. Amplification products after qPCR on a 1.5% agarose gel. Amplicons of reverse transcriptase (RT), without reverse transcriptase (-RT) and a non target control (NTC) were applied.

Images of original drafts

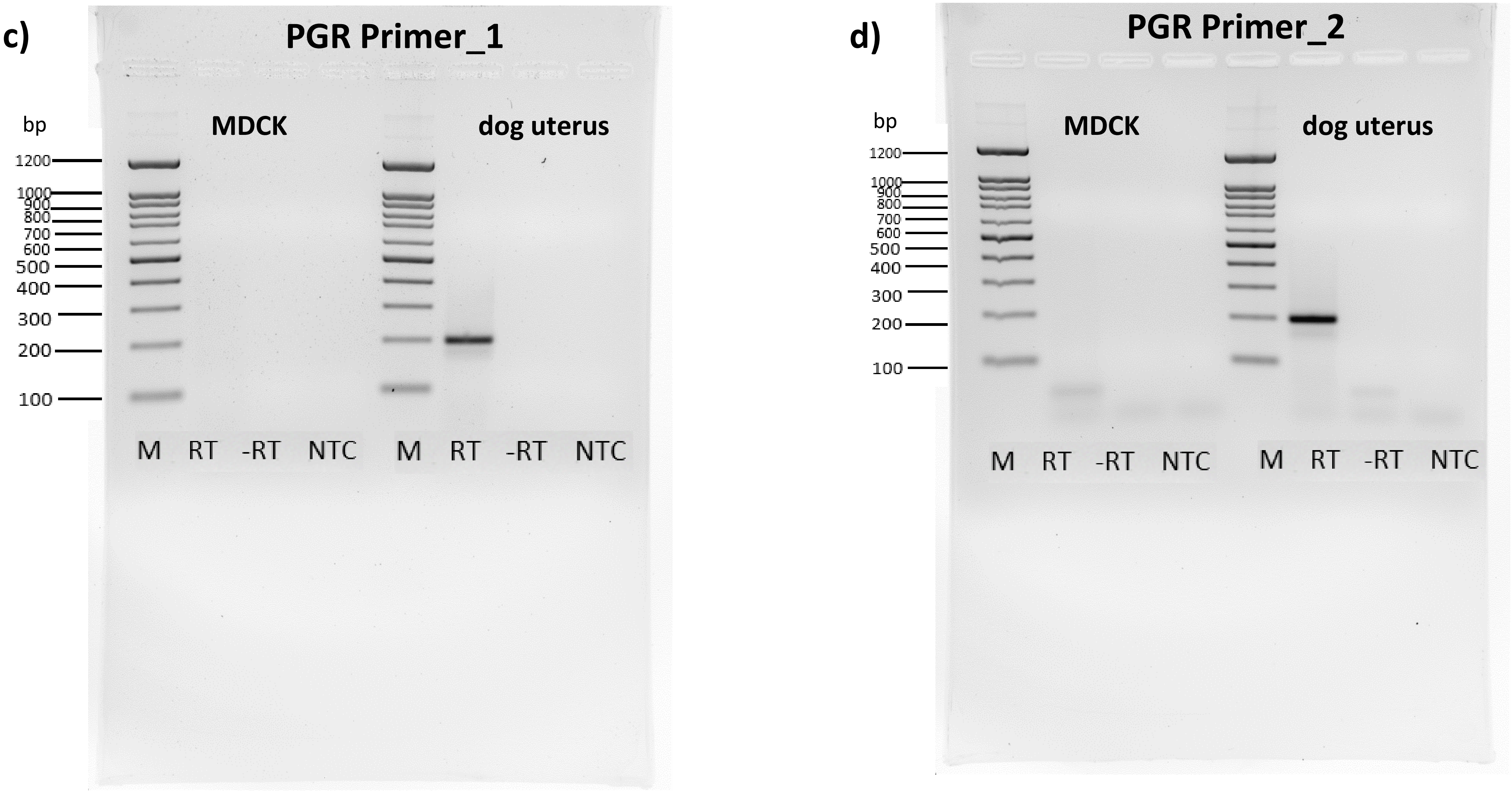

Original gel image shown in Suppl. Figure S3 A. c) Qualitative detection of PGR Primer_1 in MDCK cells (left) and dog uterus (positive control; right). Amplification products after qPCR on a 1.5% agarose gel. Amplicons of reverse transcriptase (RT), without reverse transcriptase (-RT) and a non target control (NTC) were applied. **d)** Qualitative detection of PGR Primer_2 in MDCK cells and dog uterus (positiv control). Amplification products after qPCR on a 1.5% agarose gel. Amplicons of reverse transcriptase (RT), without reverse transcriptase (-RT) and a non target control (NTC) were applied.

Images of original drafts

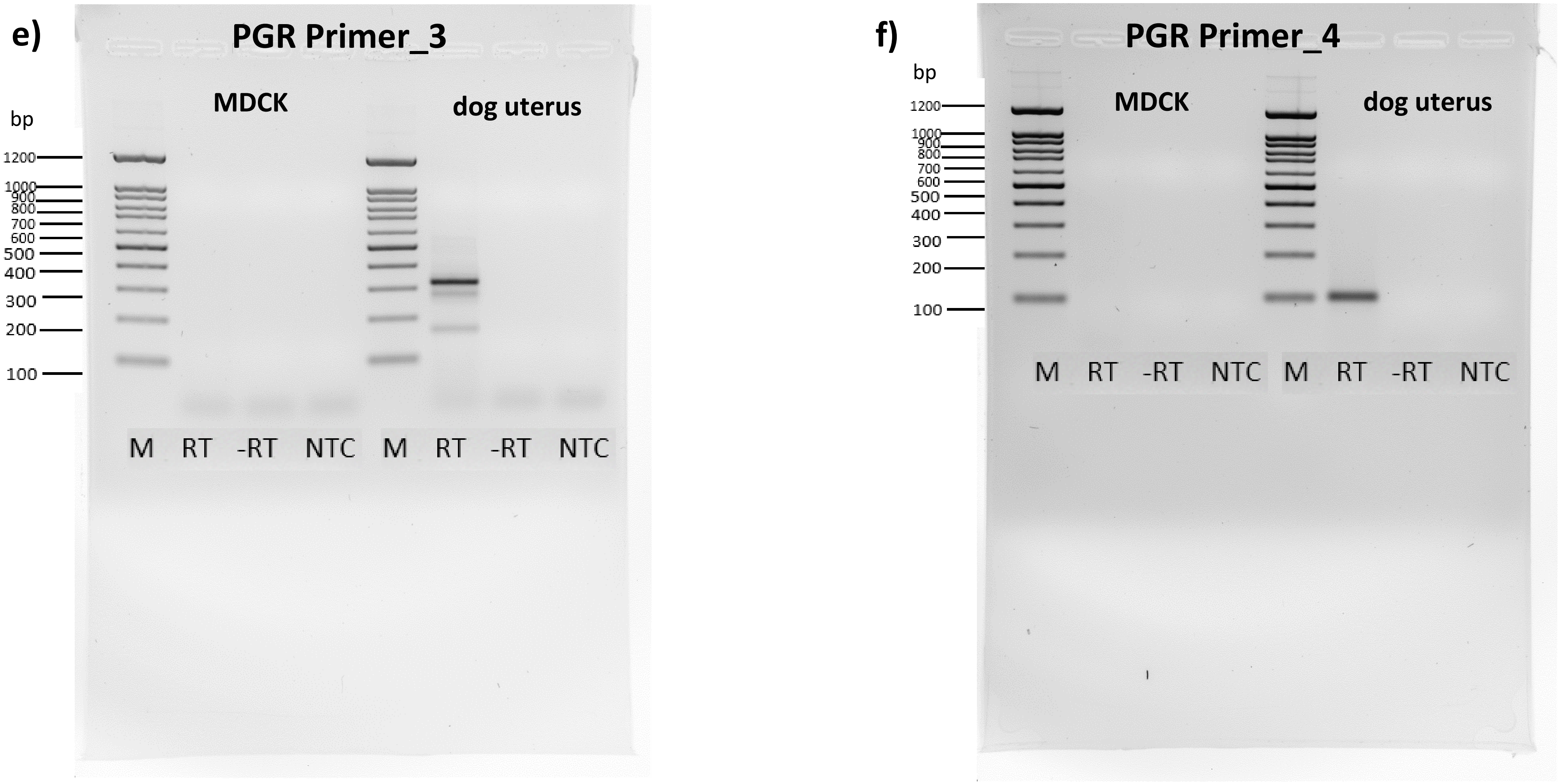

Original gel image shown in Suppl. Figure S3 A. e) Qualitative detection of PGR Primer_3 in MDCK cells (left) and dog uterus (positive control; right). Amplification products after qPCR on a 1.5% agarose gel. Amplicons of reverse transcriptase (RT), without reverse transcriptase (-RT) and a non target control (NTC) were applied. **f)** Qualitative detection of PGR Primer_4 in MDCK cells and dog uterus (positiv control). Amplification products after qPCR on a 1.5% agarose gel. Amplicons of reverse transcriptase (RT), without reverse transcriptase (-RT) and a non target control (NTC) were applied.

Images of original drafts

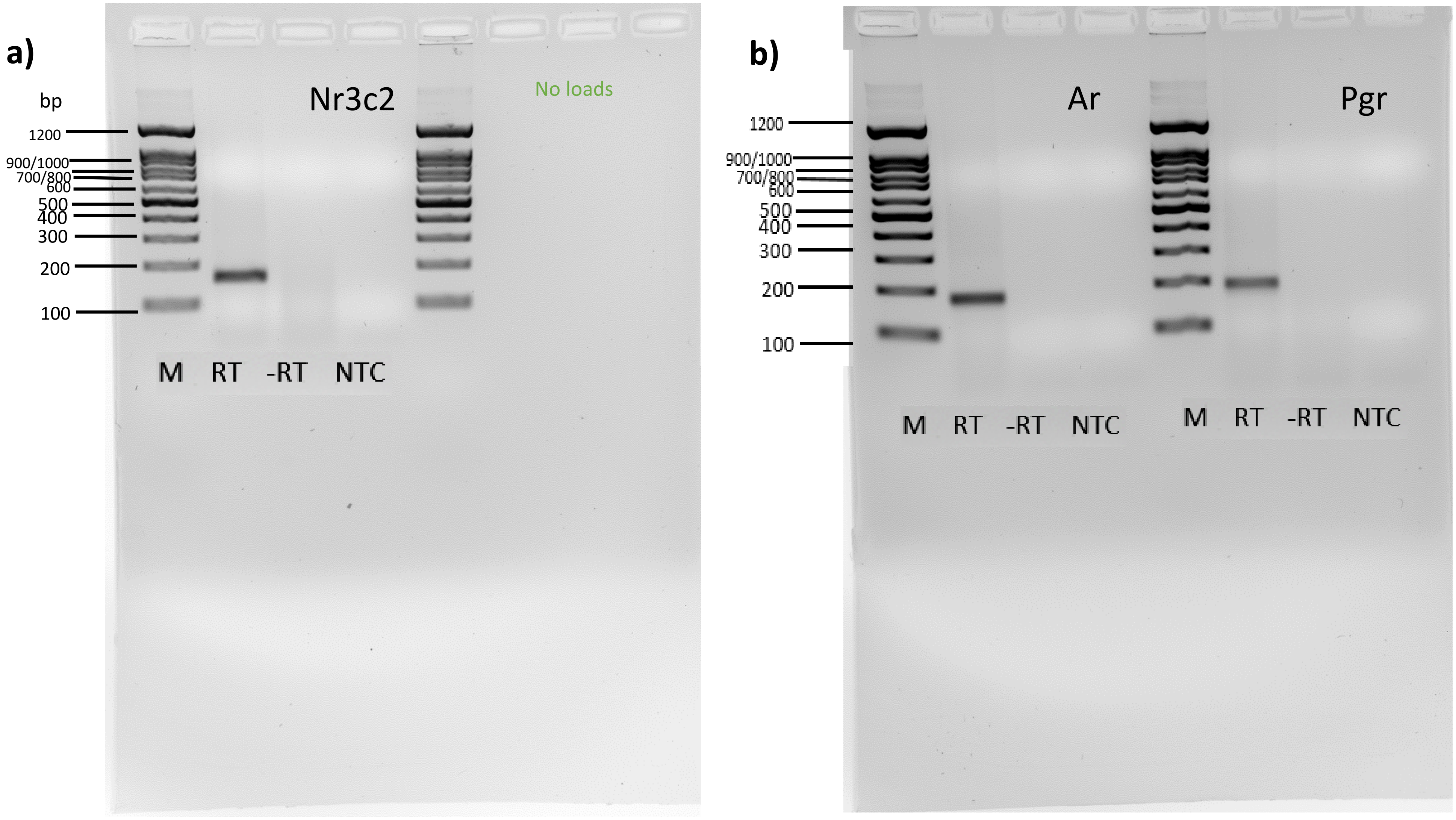

Original gel image shown in Suppl. Figure S3 B. **a)** Qualitative detection of Nr3c2 in NRK-52E cells. Amplification products after qPCR on a 1.5% agarose gel. Amplicons of reverse transcriptase (RT), without reverse transcriptase (-RT) and a non target control (NTC) were applied**. b)** Qualitative detection of Ar (left) and Pgr (right) in NRK-52E cells. Amplification products after qPCR on a 1.5% agarose gel. Amplicons of reverse transcriptase (RT), without reverse transcriptase (-RT) and a non target control (NTC) were applied.

Images of original drafts

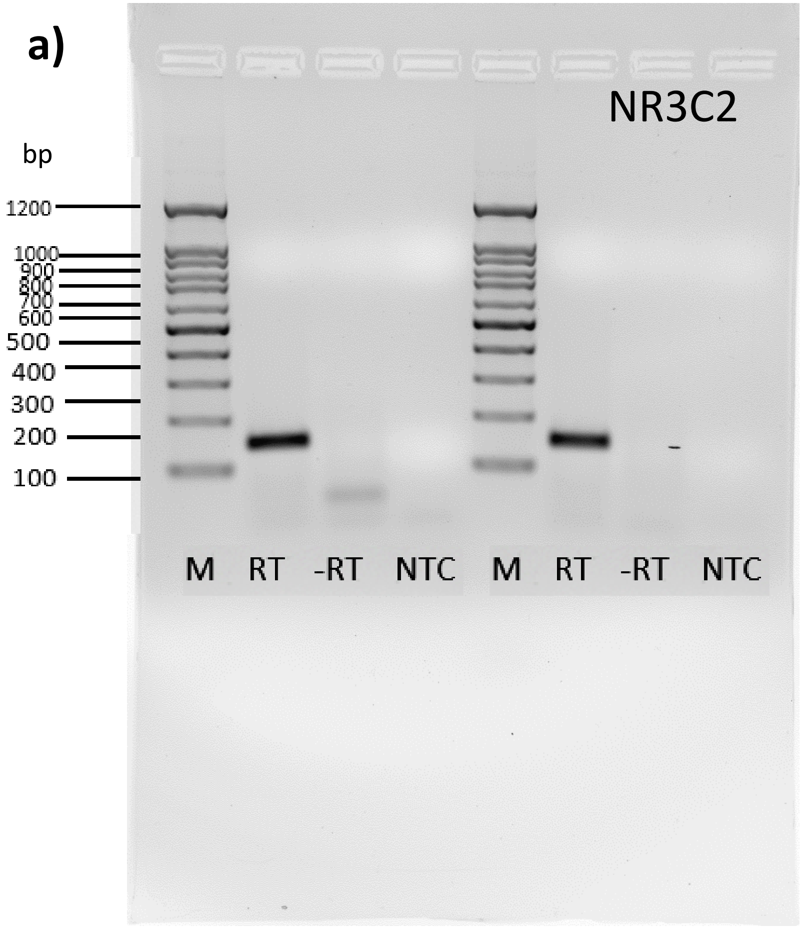

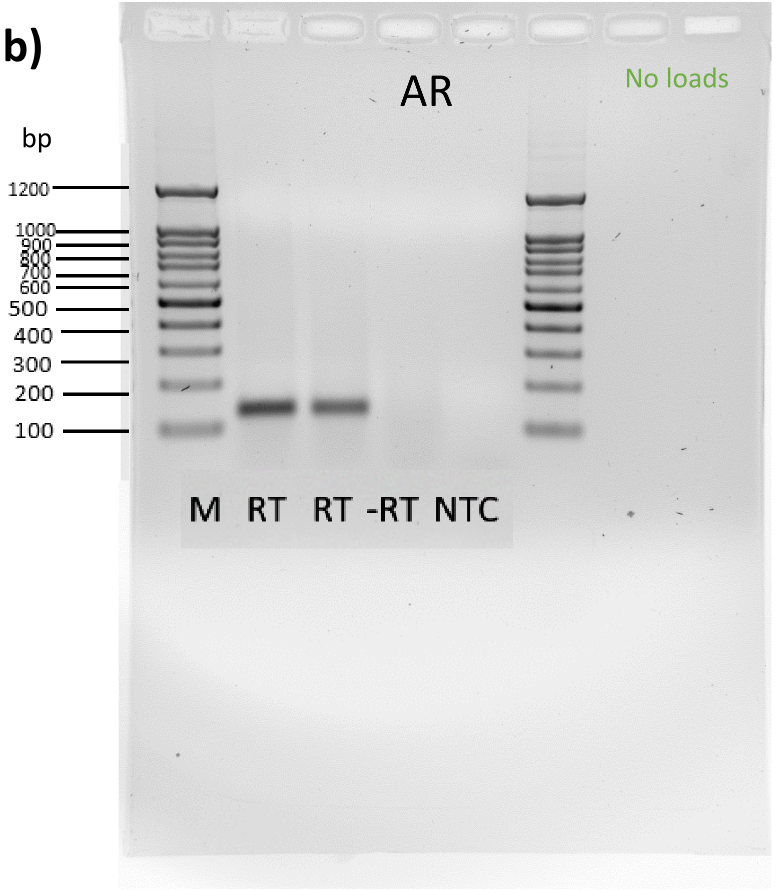

Images of original drafts

c)

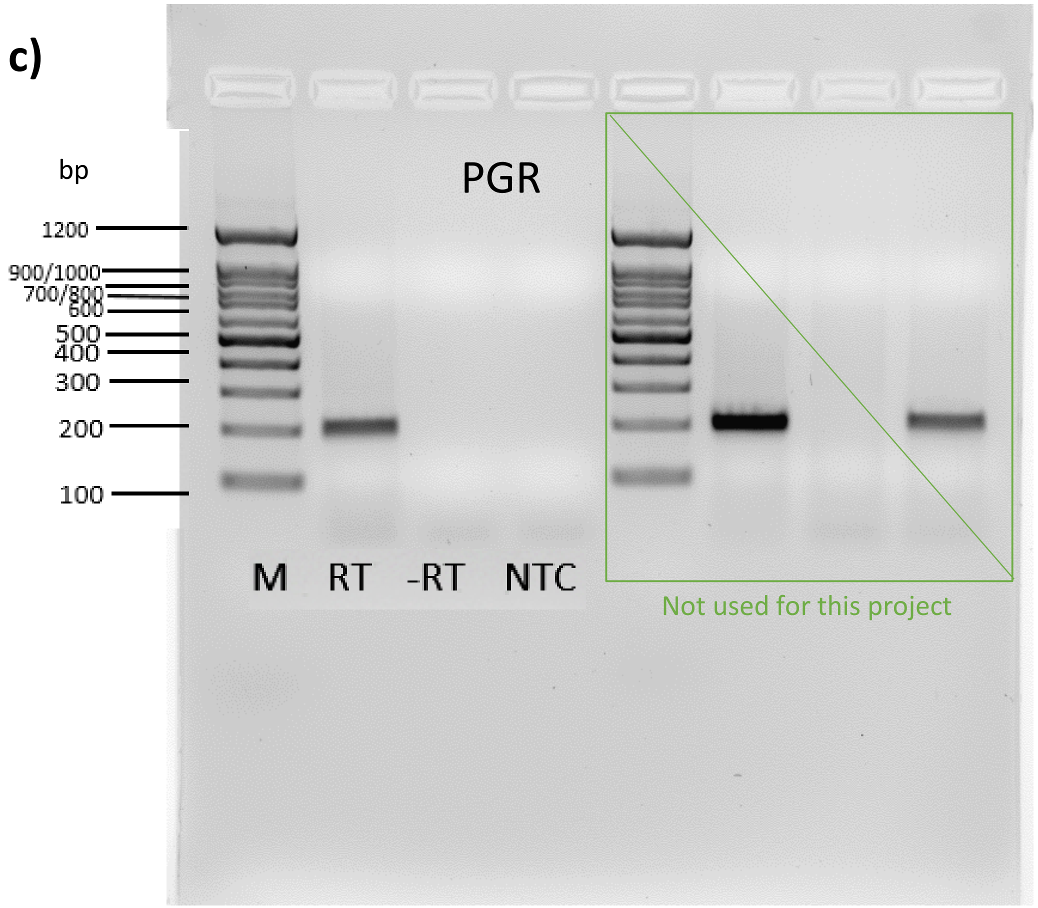

Original gel image shown in Suppl. Figure S3 C. a) Qualitative detection of NR3C2 in HK2 cells. Amplification products after qPCR on a 1.5% agarose gel. Amplicons of reverse transcriptase (RT), without reverse transcriptase (-RT) and a non target control (NTC) were applied **b)** Qualitative detection of AR in HK2 cells. Amplification products after qPCR on a 1.5% agarose gel. Amplicons of reverse transcriptase (RT), without reverse transcriptase (-RT) and a non target control (NTC) were applied **c)** Qualitative detection of PGR in HK2 cells. Amplification products after qPCR on a 1.5% agarose gel. Amplicons of reverse transcriptase (RT), without reverse transcriptase (-RT) and a non target control (NTC) were applied. Bands marked in green are not part of this project.

Images of original drafts

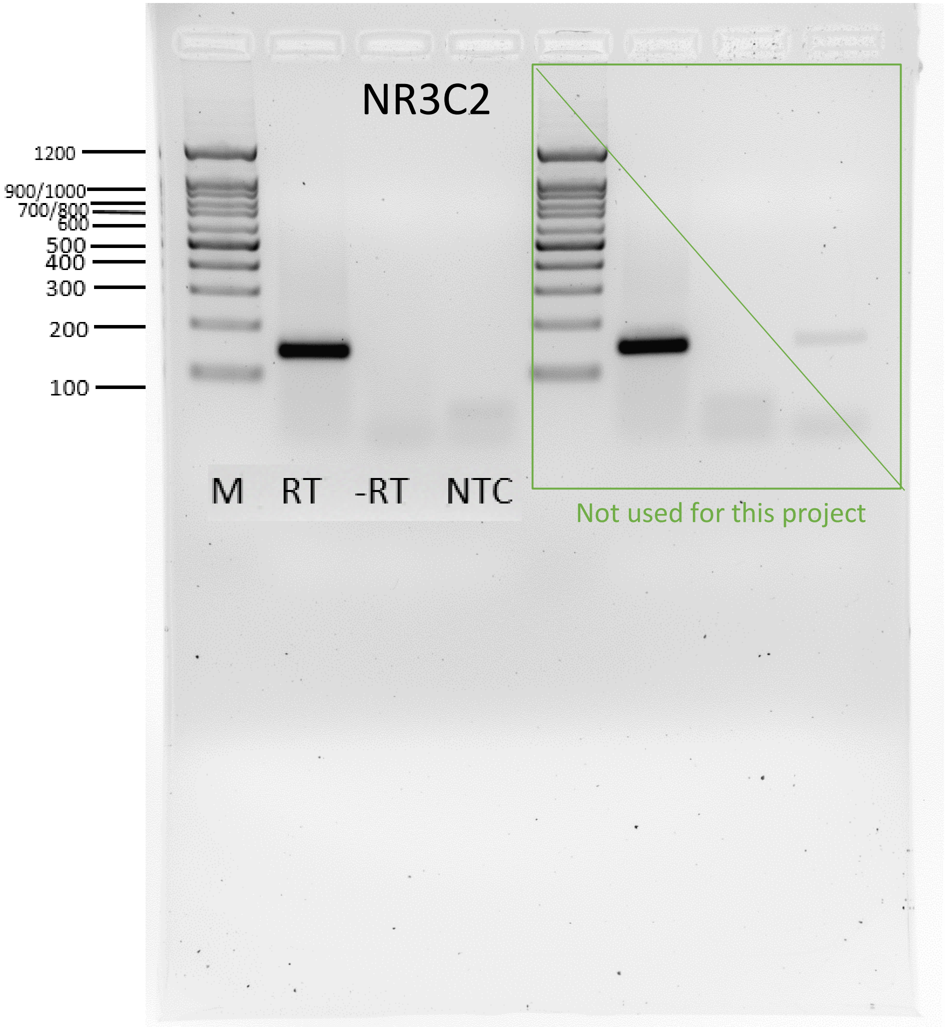

Original gel image shown in Suppl. Figure S11 A. Qualitative detection of NR3C2 in RPTEC cells. Amplification products after qPCR on a 1.5% agarose gel. Amplicons of reverse transcriptase (RT), without reverse transcriptase (-RT) and a non target control (NTC) were applied. Bands marked in green are not part of this project.
