# Supplementary material for: Regulation of Klotho Production by Mineralocorticoid Receptor Signaling in Renal Cell Lines
Source: Biomolecules. 2025 Oct 25;15(11):1509. doi: 10.3390/biom15111509 (PMC12650181; doi:10.3390/biom15111509)
Supplement: Supplementary file 1 [file biomolecules-15-01509-s001.zip › biomolecules-3623839-supplementary.pdf]

Supplementary Material

1. Supplementary Materials and Methods

1.1 Primer Efficiency Test

To determine primer efficiency, cDNA samples from rat kidney, dog uterus, and HK2 cells were diluted in a serial dilution of 1:10 to create a standard curve. Reaction mixes containing 0.25 µM (*Klotho*) or 0.5 µM (*TATA box-binding protein, TBP*) of the respective primers, 10 µl GoTaq qPCR Master Mix (Promega, Mannheim, Germany), and water were used. PCR conditions were: 95 °C for 2 min; 40 cycles of 95 °C for 10 s, primer-specific temperature for 30 s; and 72 °C for 25 s. Amplification efficiency of the qRT-PCR reactions were calculated based on the slope of the standard curve.

Primer efficiency of the following primers (5' → 3') was determined:

Klotho (*Kl*), 54 °C (rat): CAACTACATTCAAGTGGACC and CAGTAAGGTTTTCTCTTCTTGG;

Klotho (*KL*), 56 °C (dog): AAATGAAGCTCTGAAAGCC and AATGATAGAGGCCAAACTTC;

Klotho (*KL*), 59 °C (human): TGGAAACCTTAAAAGCCATCAAGC and CCACGCCTGATGCTGTAACC;

Tbp, 57 °C (rat): ACTCCTGCCACACCAGCC and GGTCAAGTTTACAGCCAAGATTCA;

TBP, 60 °C (dog): CCTATTACCCCTGCCACACC and GCTCCCGTACACACCATCTT;

TBP, 59 °C (human): TGCACAGGAGCCAAGAGTGAA and CACATCACAGCTCCCCACCA.

1.2 Evaluation of RNA Quality

RNA samples were incubated with RiboRuler RNA-loading buffer (SM1821, Thermo Fisher Scientific, Darmstadt, Germany) for 10 min at 65 °C, loaded onto a 1% agarose gel containing formaldehyde and ethidium bromide. The gel was run for 50 min at 75 V. RNA bands were visualized by the Biometra BioDoc Analyze system (Analytic Jena, Jena, Germany).

1.3 Cell Viability Assay

Cell viability was determined by 3-[4,5-dimethylthiazol-2-yl]-2,5-diphenyltetrazolium bromide (MTT) assay. MDCK, NRK-52E, and HK2 cells were cultured for 24 h and then exposed to finerenone or the respective vehicle for another 24 h. Next, cells were incubated for 1 h with 0.5 mg/mL MTT solution (Sigma-Aldrich, Schnelldorf, Germany). Absorbance was measured at 550 nm (reference wavelength 650 nm) using a microplate reader (FLUOstar Omega; BMG Labtech, Ortenberg, Germany). Cell viability of the finerenone-treated cells was expressed as the percentage of cell viability of the vehicle-treated cells.

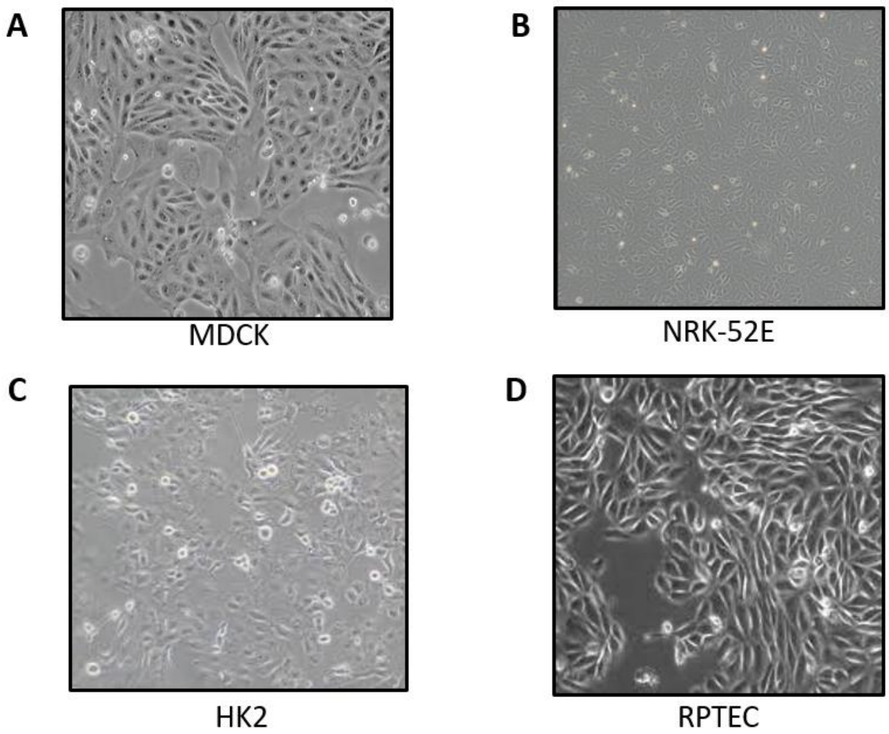

Figure S1. Light microscopy photographs of the renal cells. Photographs of untreated MDCK (**A**, taken from https://innoprot.com), NRK-52E (**B**, taken from https://taiclone.com), and HK2 (**C**, taken from https://www.ubigene.us) cells, and RPTECs (**D**, taken from https://evercyte.com).

Table S1. Additional information on the primers used.

Gene	Species	NCBI accession number	Annealing temp. [°C]	Amplification efficiency [%]	Amplicon size (bp)
Klotho (*KL*)	dog	XM_038434663.1	56	108.6	117
Klotho (*Kl*)	rat	NM_031336.2	54	98.6	129
Klotho (*KL*)	human	NM_004795.4	59	90.8	109
TBP	dog	XM_038684467.1	60	89.6	230
Tbp	rat	NM_001004198.1	57	103.9	91
TBP	human	NM_003194.5	59	87.9	132

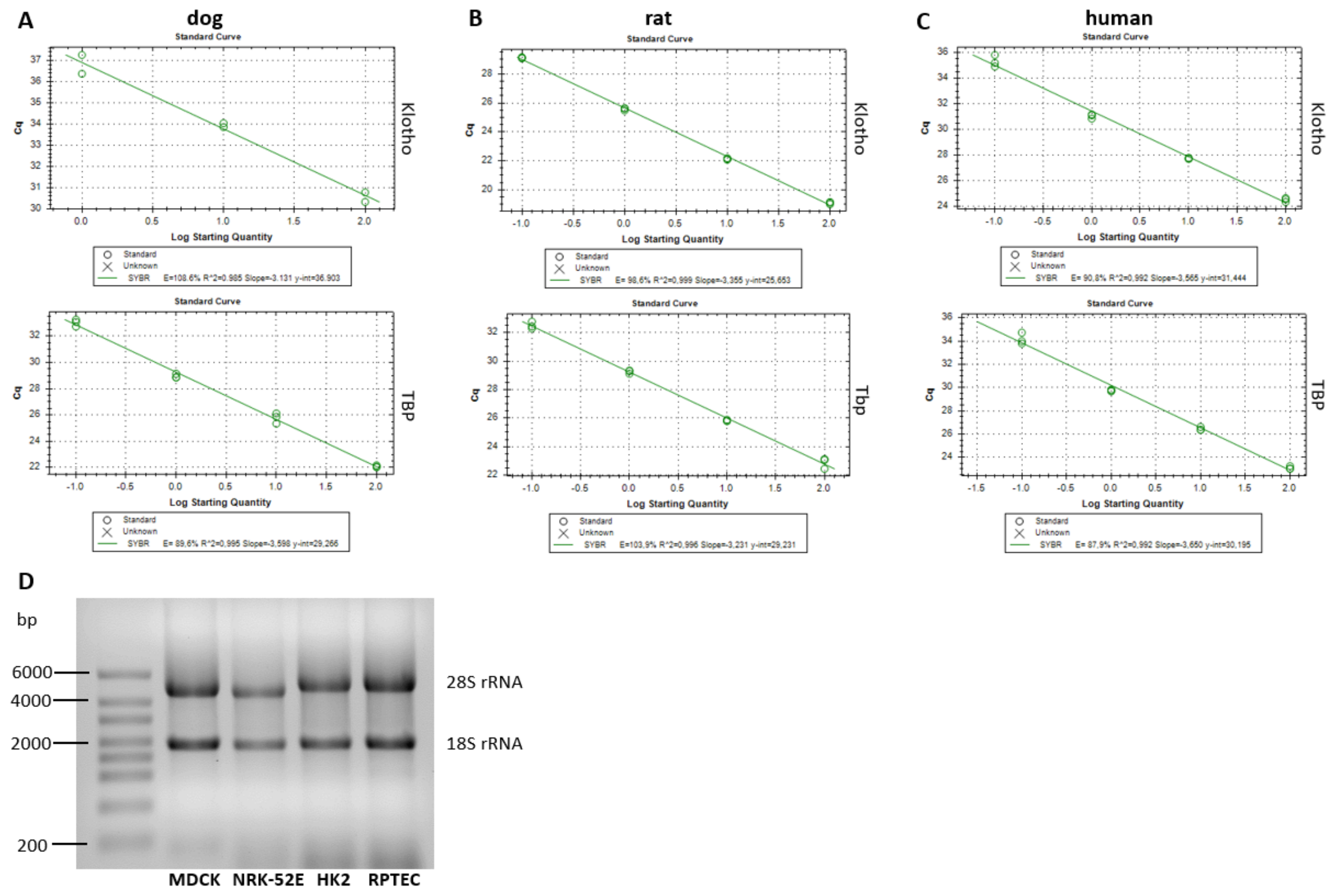

Figure S2. Evaluation of primer efficiency and RNA quality. Standard curves of *Klotho* and *Tbp* qRT-PCRs using dog (**A**), rat (**B**), and human (**C**) primers. Representative agarose gel image of RNA samples from MDCK, NRK-52E, and HK2 cells as well as RPTECs, showing the bands of 28S and 18S rRNA (**D**).

2. Supplementary Results

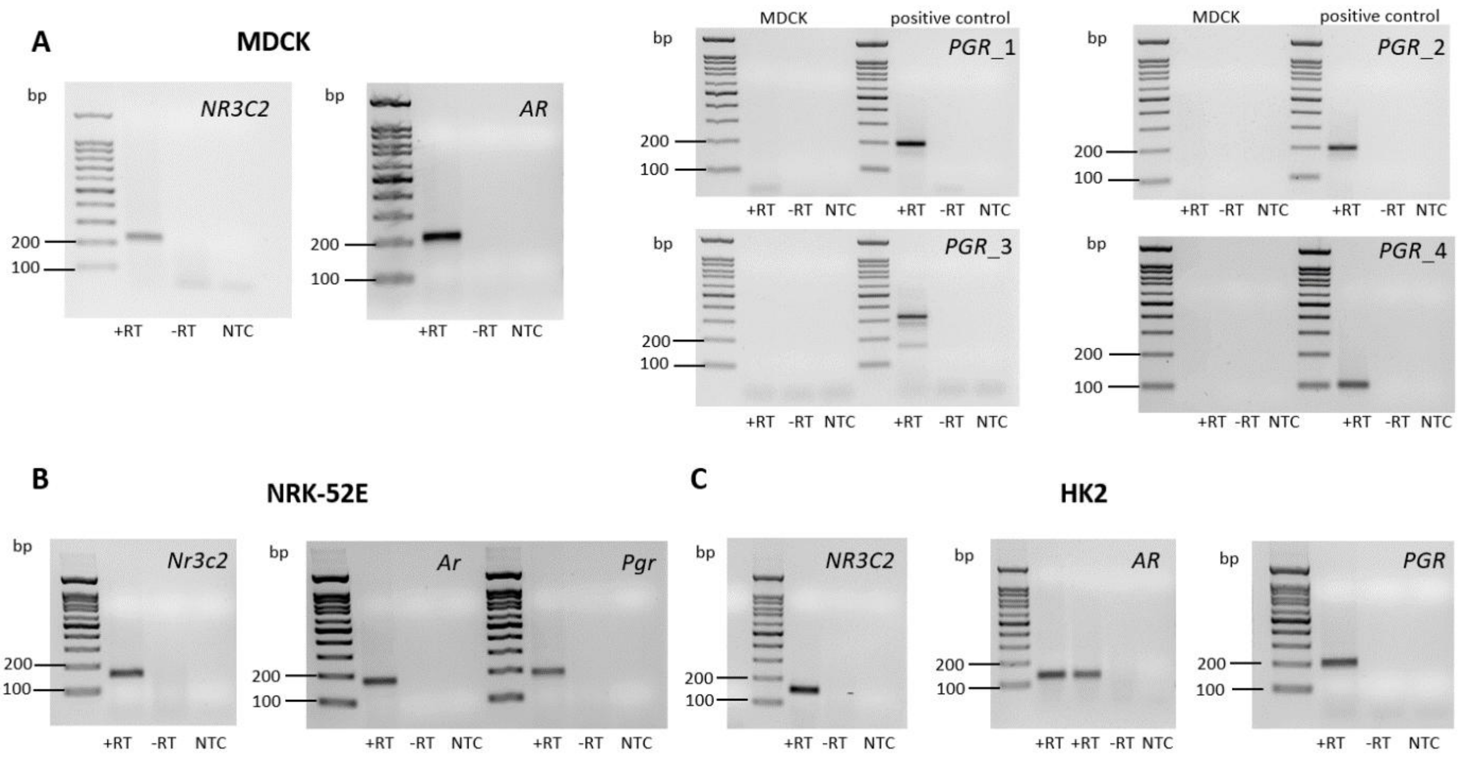

Figure S3. Qualitative expression analysis of *NR3C2*, *AR*, and *PGR* in MDCK, NRK-52E, and HK2 cells. Qualitative expression of *NR3C2* (221 bp) and *AR* (208 bp) in MDCK cells. No expression of *PGR* was detectable in MDCK cells. The positive control (dog uterus) shows expression of *PGR* with all four tested primers at 194 bp (*PGR*_1), 195 bp (*PGR*_2), 317 bp (*PGR*_3), and 97 bp (*PGR*_4) (**A**). Qualitative expression of *Nr3c2* (160 bp), *Ar* (187 bp), and *Pgr* (176 bp) in NRK-52E cells (**B**). Qualitative expression of *NR3C2* (148 bp), *AR* (131 bp), and *PGR* (198 bp) in HK2 cells (**C**). Bp, base pairs; NTC, no-template control; -RT, control without reverse transcriptase.

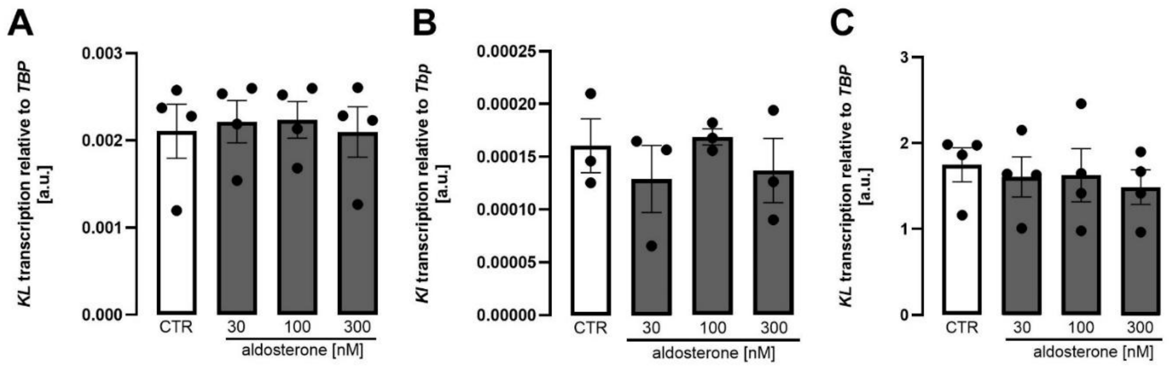

Figure S4. Aldosterone did not significantly affect *Klotho* expression in MDCK, NRK-52E, and HK2 cells cultured in growth medium supplemented with charcoal-stripped fetal bovine serum. Arithmetic means ± SEM of rel. *Klotho* (*KL*) gene expression in MDCK (**A**, n=4), NRK-52E (**B**, n=3), and HK2 (**C**, n=4) cells treated with or without aldosterone for 24 h. Cells were seeded and grown in growth medium containing charcoal-stripped FBS. One-way ANOVA followed by Dunnett’s multiple comparisons test. A.u., arbitrary units; FBS, fetal bovine serum.

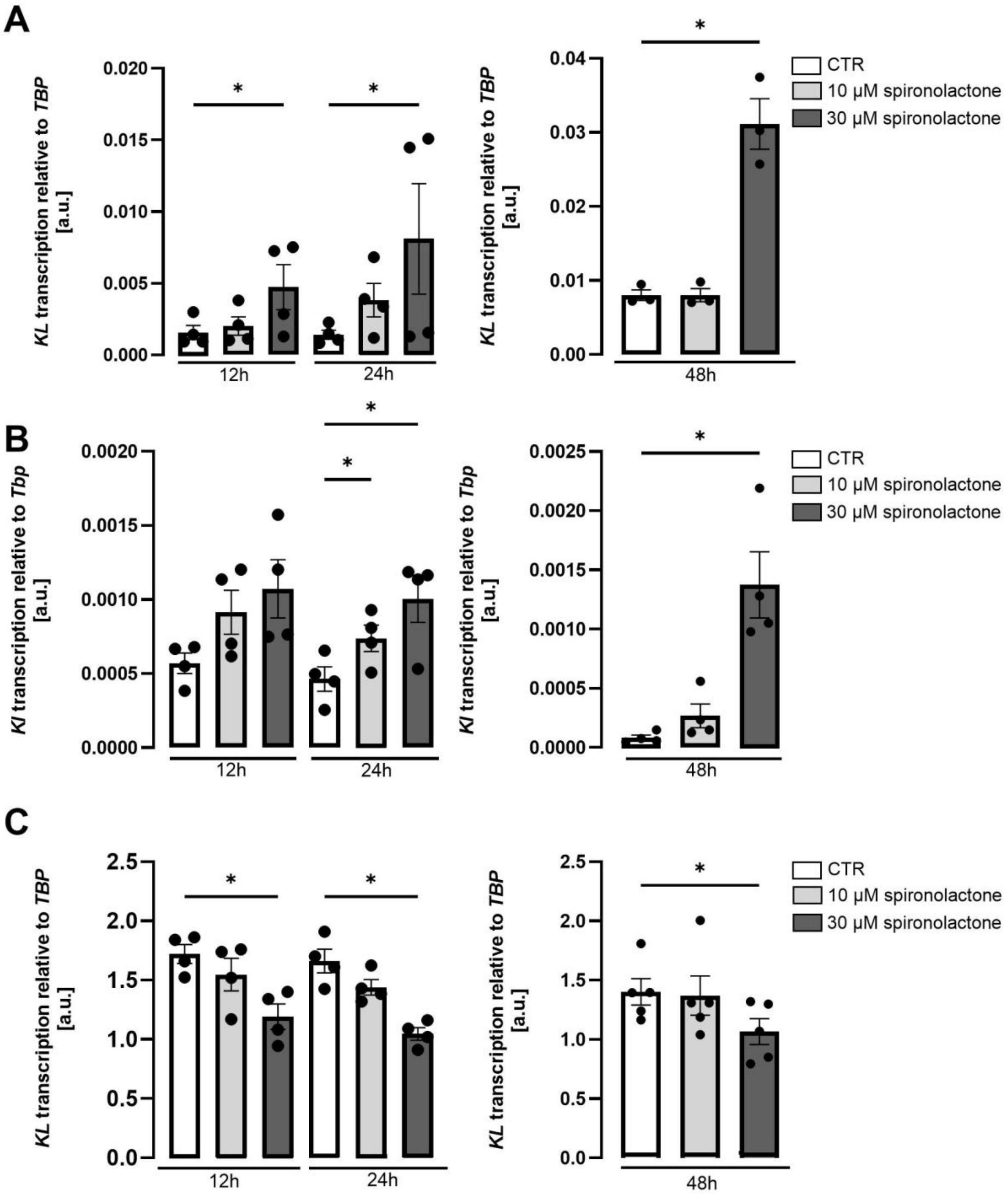

Figure S5. Time dependence of the spironolactone effect on *Klotho* expression in MDCK, NRK-52E, and HK2 cells. Arithmetic means ± SEM of rel. *Klotho* gene expression in MDCK (**A**, 12 and 24 h: n=4; 48 h: n=3), NRK-52E (**B**, 12 and 24 h: n=4; 48 h: n=4), and HK2 (**C**, 12 and 24 h: n=4; 48 h: n=5) cells treated with or without spironolactone for 12, 24 or 48 h. One-way ANOVA followed by Dunnett's multiple comparisons or Friedman test; *$p < 0.05$. A.u., arbitrary units.

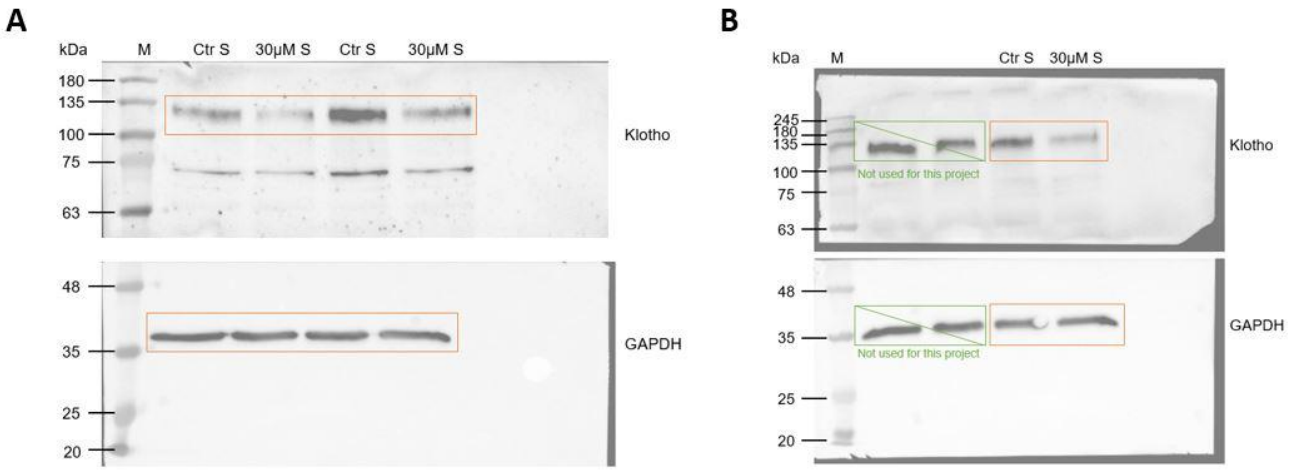

Figure S6. Original Western blot shown in Fig. 2D. Bands marked in orange were used for analysis. HK2 cells were treated for 24 h with or without 30 µM spironolactone (S). GAPDH was used as loading control. Membrane was cut between 63 and 48 kDa (**A**). Original Western blot not shown in the article. Bands marked in orange were used for analysis, but are not shown in Fig. 2D. HK2 cells were treated for 24 h with or without 30 µM spironolactone (S). GAPDH was used as a loading control. Lanes marked in green are not part of the work presented here and have no relevance for the evaluation and assessment. Membrane was cut between 63 and 48 kDa (**B**). M, marker.

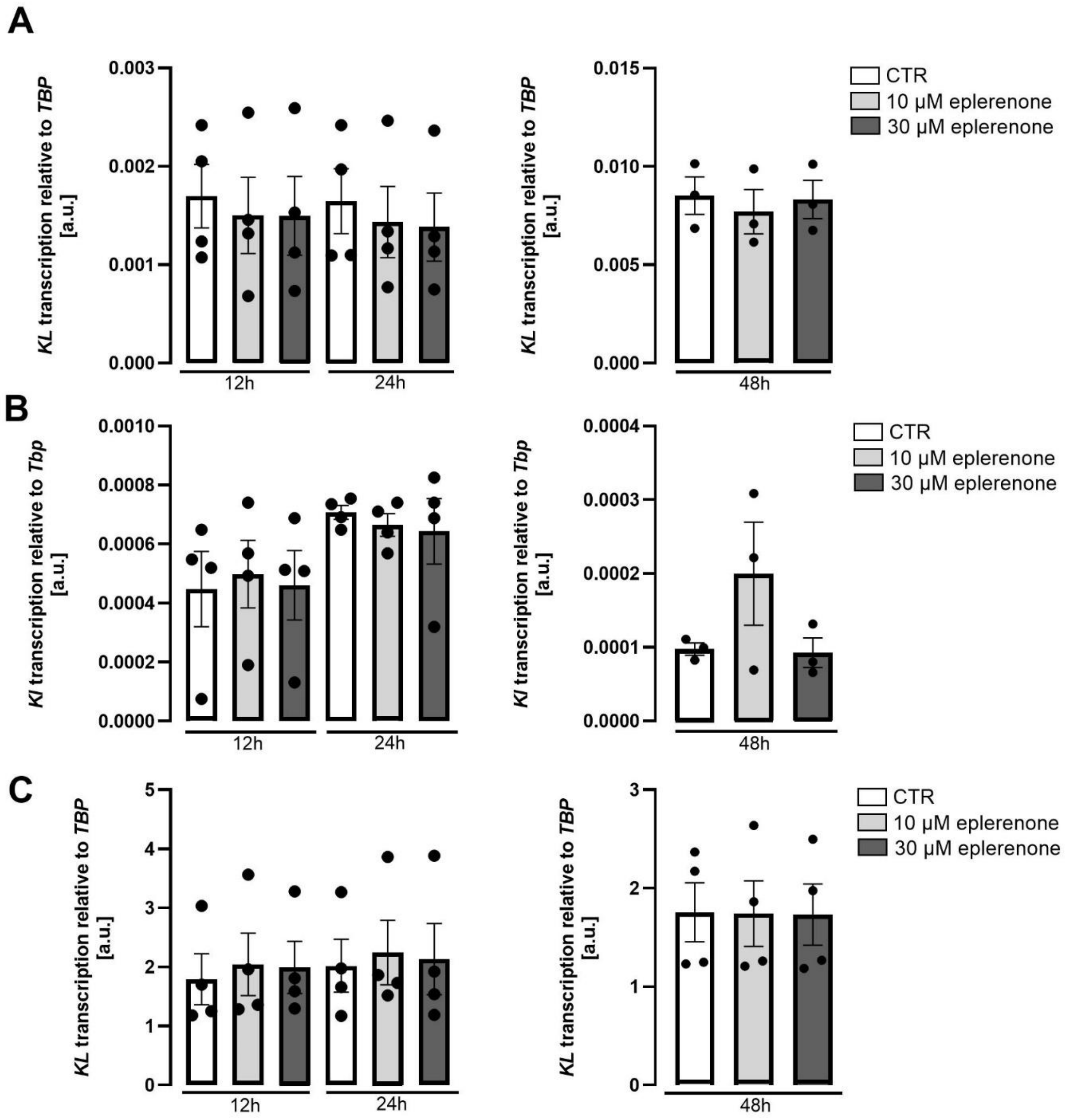

Figure S7. Time dependence of the eplerenone effect on *Klotho* expression in MDCK, NRK-52E, and HK2 cells. Arithmetic means ± SEM of rel. *Klotho* gene expression in MDCK (**A**, 12 and 24 h: n=4; 48 h: n=3), NRK-52E (**B**, 12 and 24 h: n=4; 48 h: n=3), and HK2 (**C**, 12 and 24 h: n=4; 48 h: n=4) cells treated with or without eplerenone for 12, 24 or 48 h. One-way ANOVA followed by Dunnett's multiple comparisons test; $*p < 0.05$. A.u., arbitrary units.

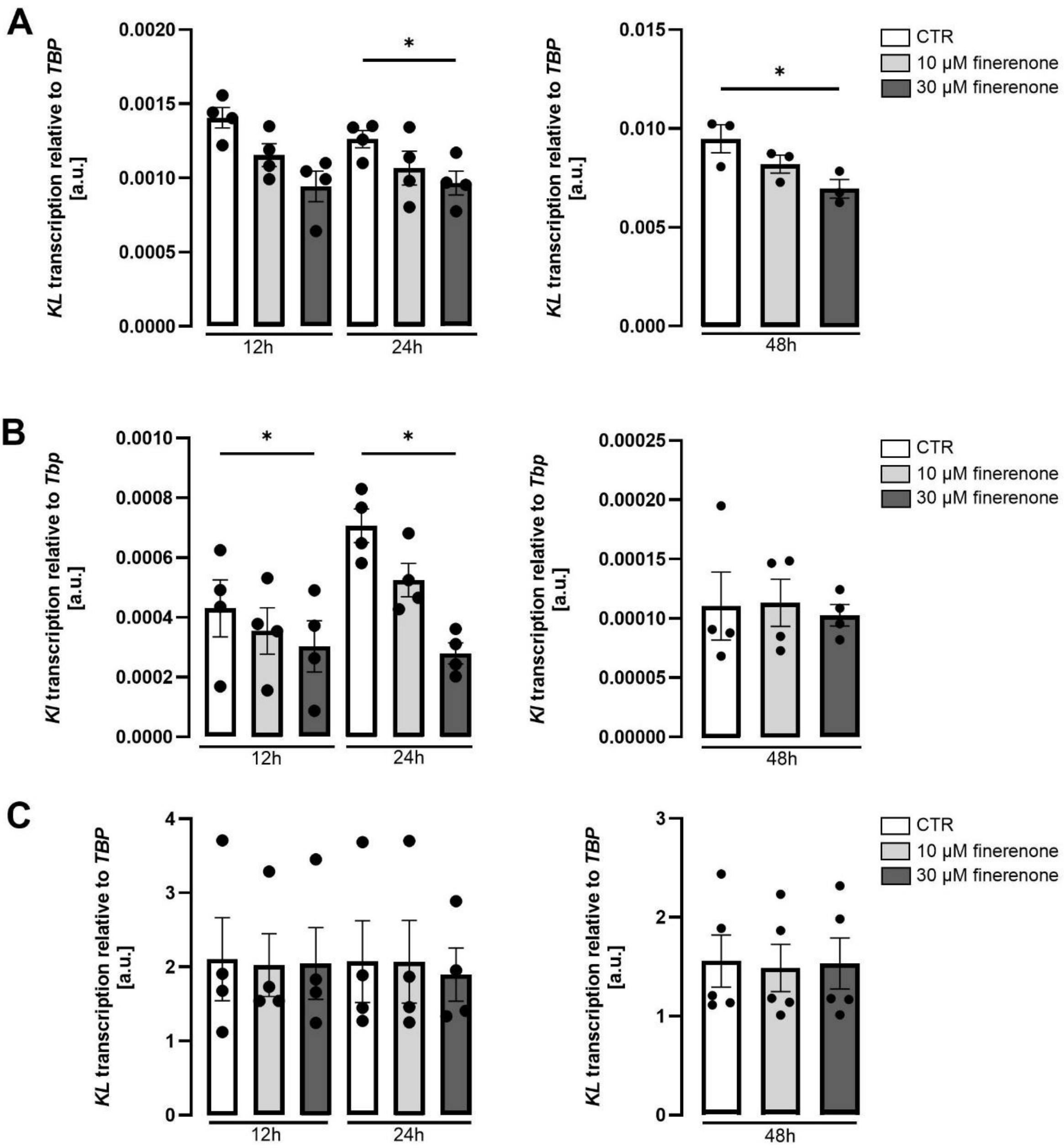

Figure S8. Time dependence of the finerenone effect on *Klotho* expression in MDCK, NRK-52E, and HK2 cells. Arithmetic means ± SEM of rel. *Klotho* gene expression in MDCK (**A**, 12 and 24 h: n=4; 48 h: n=3), NRK-52E (**B**, 12 and 24 h: n=4; 48 h: n=4), and HK2 (**C**, 12 and 24 h: n=4; 48 h: n=5) cells treated with or without finerenone for 12, 24 or 48 h. One-way ANOVA followed by Dunnett's multiple comparisons test; *$p < 0.05$. A.u., arbitrary units.

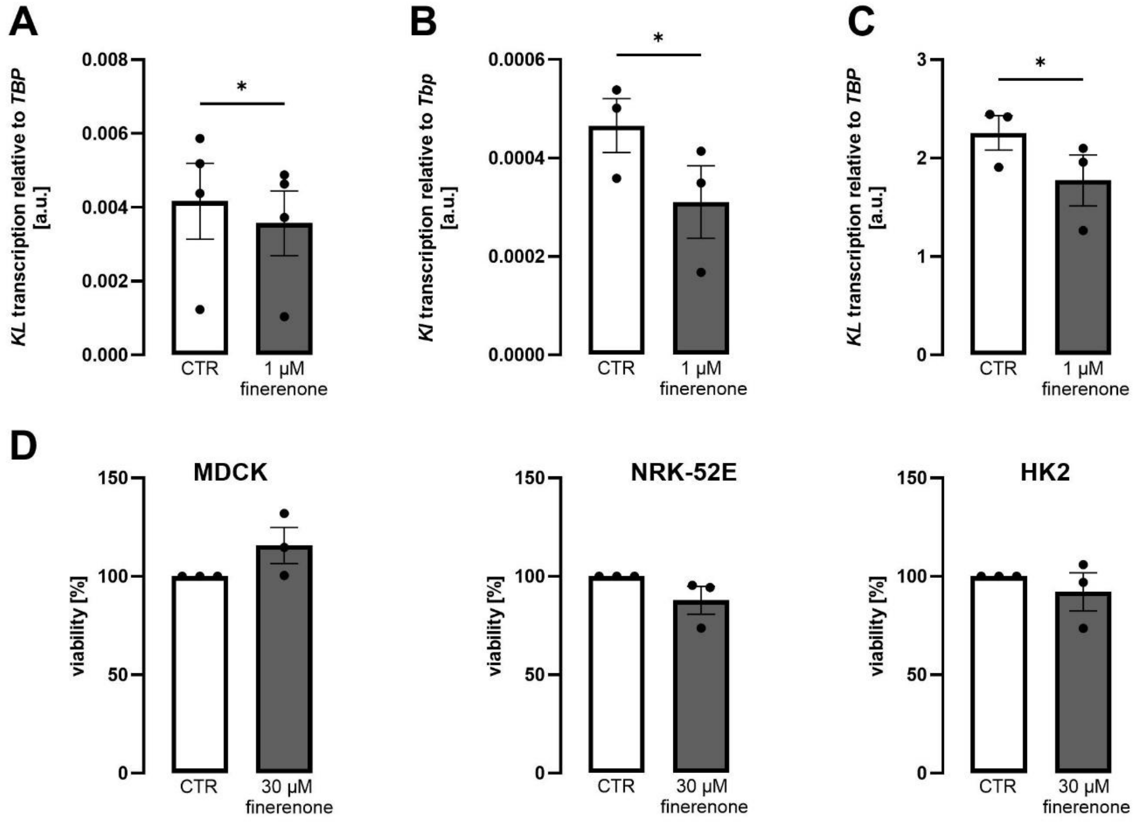

Figure S9. Low-dose finerenone reduced *Klotho* expression in MDCK, NRK-52E, and HK2 cells. Arithmetic means ± SEM of rel. *Klotho* gene expression in MDCK (**A**, n=4), NRK-52E (**B**, n=3), and HK2 (**C**, n=3) cells treated with or without finerenone for 24 h. High-dose finerenone treatment for 24 h did not affect cell viability of all three cell lines examined (**D**, n=3). Two-tailed paired *t*-test; *$p < 0.05$. A.u., arbitrary units.

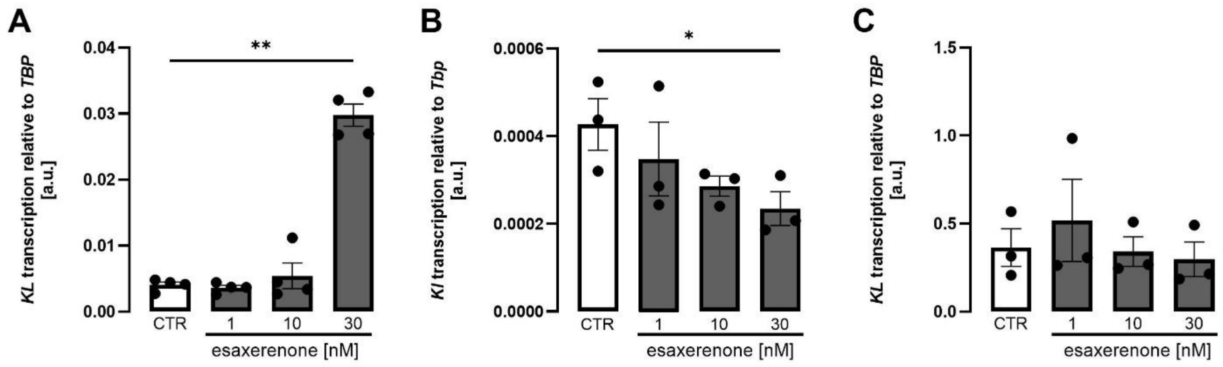

Figure S10. Esaxerenone increased *Klotho* expression in MDCK cells, decreased *Klotho* in NRK-52E cells, and had no significant effect on *Klotho* in HK2 cells. Arithmetic means ± SEM of rel. *Klotho* gene expression in MDCK (**A**, n=4), NRK-52E (**B**, n=3), and HK2 (**C**, n=3) cells treated with or without esaxerenone for 24 h. One-way ANOVA followed by Dunnett's multiple comparisons test; *$p < 0.05$, **$p < 0.01$. A.u., arbitrary units.

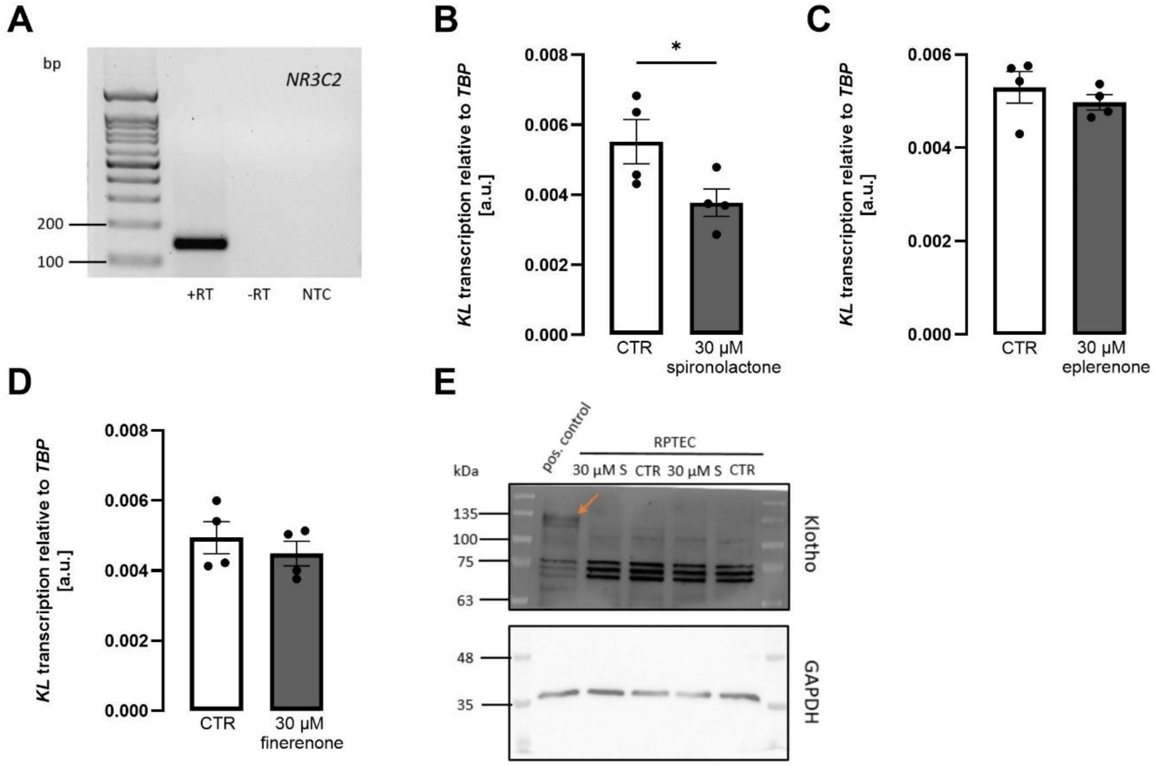

Figure S11. Spironolactone reduced *Klotho* mRNA transcription in RPTECs. Qualitative gene expression of *NR3C2* (148 bp) in RPTECs (**A**). Arithmetic means ± SEM of rel. *Klotho* gene expression in RPTECs treated with or without spironolactone (**B**, n=4), eplerenone (**C**, n=4), or finerenone (**D**, n=4) for 24 h. Original Western blot of RPTECs treated with or without spironolactone (S) for 24 h. Untreated HK2 cells were used as a positive control for Klotho protein: expected band at approximately 120 kDa (marked by an arrow) (**E**). The original Western blot image is shown in Fig. S12. Two-tailed paired *t*-test; $*p < 0.05$. A.u., abitrary units; bp, base pairs; NTC, no-template control; -RT, control without reverse transcriptase.

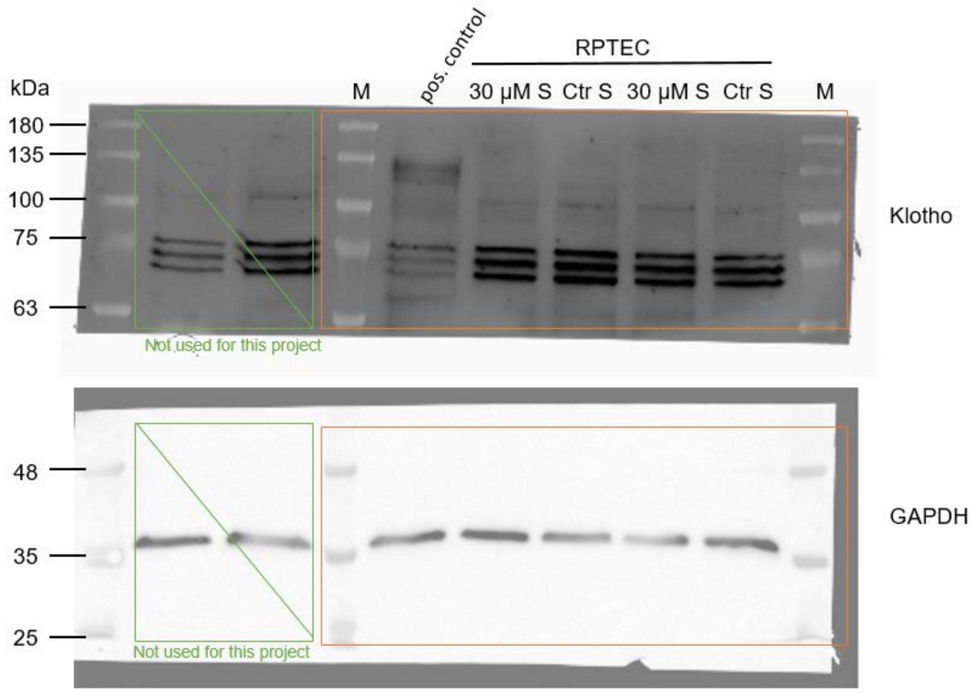

Figure S12. Original Western blot shown in Fig. S11E. Bands marked in orange were shown in Fig. S11E. RPTECs were treated for 24 h with or without 30 µM spironolactone (S). Membrane was cut between 63 and 48 kDa, and GAPDH was used as loading control. Lanes marked in green are not part of the work presented here. M, marker.
